# Functional *in vitro* diversity of an intrinsically disordered plant protein during freeze–thawing is encoded by its structural plasticity

**DOI:** 10.1002/pro.4989

**Published:** 2024-04-24

**Authors:** Itzell Hernández‐Sánchez, Tobias Rindfleisch, Jessica Alpers, Martin Dulle, Christopher J. Garvey, Patrick Knox‐Brown, Markus S. Miettinen, Gergely Nagy, Julio M. Pusterla, Agata Rekas, Keyun Shou, Andreas M. Stadler, Dirk Walther, Martin Wolff, Ellen Zuther, Anja Thalhammer

**Affiliations:** ^1^ Max‐Planck Institute of Molecular Plant Physiology Potsdam Germany; ^2^ Physical Biochemistry University of Potsdam Potsdam Germany; ^3^ Department of Chemistry University of Bergen Bergen Norway; ^4^ Computational Biology Unit, Department of Informatics University of Bergen Bergen Norway; ^5^ Jülich Centre for Neutron Science (JCNS‐1) and Institute of Biological Information Processing (IBI‐8: Neutron Scattering and Biological Matter) Forschungszentrum Jülich GmbH Jülich Germany; ^6^ Heinz Maier‐Leibnitz Zentrum (MLZ) Technische Universität München Garching Germany; ^7^ Department of Theory and Bio‐Systems Max Planck Institute of Colloids and Interfaces Potsdam Germany; ^8^ Neutron Scattering Division Oak Ridge National Laboratory Oak Ridge Tennessee USA; ^9^ Australian Nuclear Science and Technology Organization (ANSTO) Kirrawee New South Wales Australia; ^10^ Institute of Physical Chemistry, RWTH Aachen University Aachen Germany; ^11^ Present address: Center for Desert Agriculture, Biological and Environmental Science and Engineering Division King Abdullah University of Science and Technology (KAUST) Thuwal Saudi Arabia; ^12^ Present address: Department of Discovery Pharmaceutical Sciences Merck & Co., Inc. South San Francisco California USA; ^13^ Present address: Center of Artificial Intelligence in Public Health Research (ZKI‐PH) Robert Koch Institute Berlin Germany

**Keywords:** freezing tolerance, functional plasticity, intrinsically disordered protein, late embryogenesis abundant protein, self‐assembly

## Abstract

Intrinsically disordered late embryogenesis abundant (LEA) proteins play a central role in the tolerance of plants and other organisms to dehydration brought upon, for example, by freezing temperatures, high salt concentration, drought or desiccation, and many LEA proteins have been found to stabilize dehydration‐sensitive cellular structures. Their conformational ensembles are highly sensitive to the environment, allowing them to undergo conformational changes and adopt ordered secondary and quaternary structures and to participate in formation of membraneless organelles. In an interdisciplinary approach, we discovered how the functional diversity of the *Arabidopsis thaliana* LEA protein COR15A found *in vitro* is encoded in its structural repertoire, with the stabilization of membranes being achieved at the level of secondary structure and the stabilization of enzymes accomplished by the formation of oligomeric complexes. We provide molecular details on intra‐ and inter‐monomeric helix–helix interactions, demonstrate how oligomerization is driven by an α‐helical molecular recognition feature (α‐MoRF) and provide a rationale that the formation of noncanonical, loosely packed, right‐handed coiled‐coils might be a recurring theme for homo‐ and hetero‐oligomerization of LEA proteins.

## 1 INTRODUCTION

Many plants can withstand periods of low to essentially no water. This ranges from the rather mild dehydration cells experience, for example, during freezing to almost full desiccation in seeds (Bartels & Salamini, 2001). Plants have evolved multiple mechanisms to cope with water scarcity, including the expression of late embryogenesis abundant (LEA) genes. These transcripts and the according proteins accumulate in most plant species in response to various environmental conditions resulting in mild to severe cellular water limitation brought upon, for example, by freezing temperatures, high salt concentrations, drought or actual desiccation, and their expression is mostly regulated via the phytohormone abscisic acid (e.g. Chen et al., 2021; Cheng et al., 2021; Ding et al., 2021; Gechev et al., 2021; He et al., 2021; Jin et al., 2019; Li et al., 2022; Liu et al., 2019), which is a major player in controlling abiotic stress regulation (Tuteja, 2007). Additionally, LEA protein accumulation was described in several anhydrobiotic non‐plant organisms (Hand et al., 2011). Although there is a general consensus that LEA proteins play vital roles in the tolerance of plants to all these diverse conditions of water limitation, their molecular functional modes remain largely elusive, as *in planta* studies are facing major challenges in the identification of molecular *in vivo* targets due to weak target binding and of unfavorable water‐depleted experimental *in vivo* conditions. At the cellular level, osmotic stress is a consequence that is common to the different environmental conditions under which LEA proteins accumulate, conferred either by actual water limitation during drought, the accumulation of ions at high salinity or an osmotic gradient resulting from ice formation in the apoplast (Verslues et al., 2006). This negatively affects membrane organization and protein integrity (Krasensky & Jonak, 2012) and particularly in effects on the membrane's phase behavior and the preservation of the lamellar fluid phase, the effects of slow cooling and drying are considered equivalent (Garvey et al., 2013). A large body of *in vitro* evidence suggests that LEA proteins might function in the stabilization of such dehydration‐sensitive cellular structures like membranes (reviewed in Hernández‐Sánchez et al., 2022), proteins (reviewed in Battaglia et al., 2008, Tunnacliffe et al., 2010, and Dirk et al., 2020), or nucleic acids (Boddington & Graether, 2019; Hara et al., 2009; Kushwaha et al., 2013). Along these lines, several gain and loss of function studies reveal their importance in protecting plants during periods of water deficit (reviewed in Hernández‐Sánchez et al., 2022). One of the few LEA proteins with known physiological function is COld Regulated (COR)15A from *Arabidopsis thaliana*, which accumulates in the chloroplast stroma during cold acclimation, and acts by stabilizing chloroplast and plasma membranes during freezing *in vivo* (Thalhammer et al., 2014).

While most LEA proteins are intrinsically disordered when fully hydrated *in vitro*, their structural ensembles often respond to a reduction of water availability (reviewed in Hernández‐Sánchez et al., 2022). *In vitro*, for COR15A, this results in the stabilization of two amphipathic α‐helices that interact with membranes via their hydrophobic faces (Bremer, Kent, et al., 2017; Navarro‐Retamal et al., 2016, 2018; Sowemimo et al., 2019), and in the formation of oligomers (Shou et al., 2019). Also, *in vivo* evidence for oligomerization of LEA proteins in general (reviewed in Hernández‐Sánchez et al., 2022) and COR15A (Nakayama et al., 2007) in particular was presented previously. However, oligomerization of COR15A *in vivo* was assessed by experimental methods severely impairing the conformational flexibility inherent to an intrinsically disordered protein (IDP). Crucially, the molecular details of COR15A oligomers and, even more importantly, their functionality remains yet unknown. Oligomers might constitute the functionally active conformation, but oligomerization could alternatively represent an elegant way to scavenge monomeric COR15A to reduce potentially unshielded unfavorable hydrophobic surfaces.

Our study adds experimental evidence for the formation of COR15A homo‐oligomers under conditions suitable to reflect the dynamic nature of COR15A oligomerization without changing the equilibrium between monomers and potential oligomers and additionally demonstrates hetero‐oligomerization with its homolog COR15B *in planta*. We investigate the functional relevance of COR15A oligomers *in vivo* and *in vitro*. Finally, we provide insights into the molecular details of COR15A oligomers and into the interplay between the stabilization of secondary structure and the establishment of inter‐ and intra‐monomeric interactions derived from *in vitro* and *in silico* approaches. Our study uniquely combines physiological relevance, a high resolution of molecular detail and insights into dynamic conformational processes by integrating computational studies and experimental approaches *in vitro* and *in planta*.

## 2 RESULTS

### 2.1 Protein crowding drives self‐assembly of COR15A *in vitro* and COR15A forms homo‐oligomers and hetero‐oligomerizes with its homolog COR15B *in planta*

**FIGURE 1:**
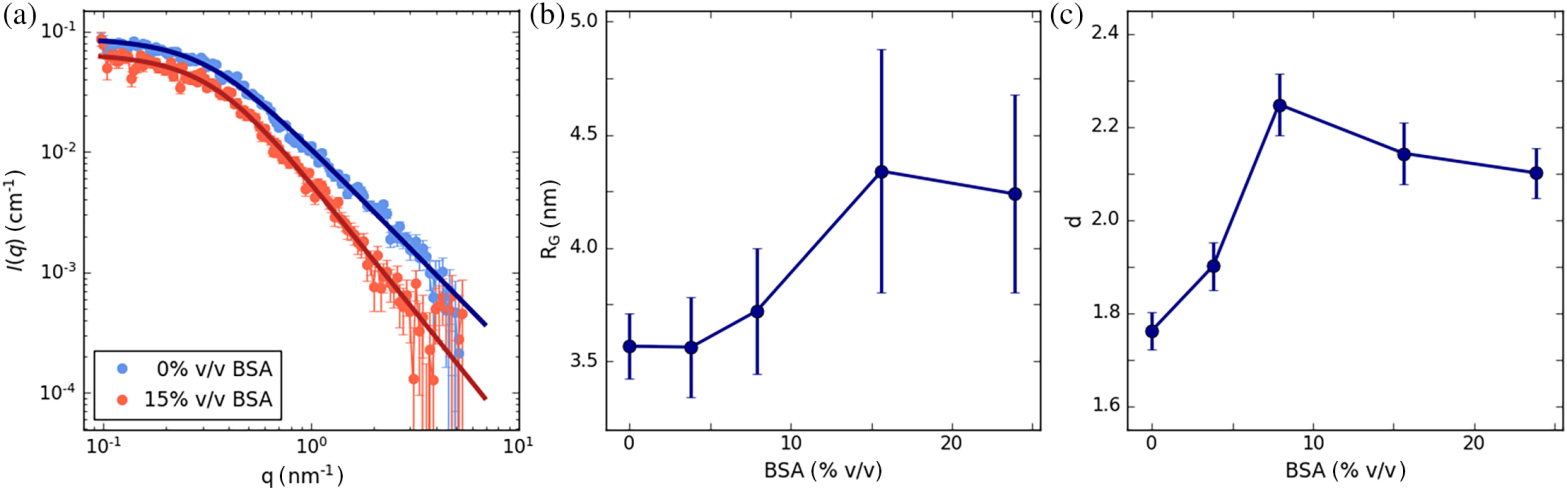
Molecular crowding induced conformational changes of COR15A were studied in increasing concentrations of the proteinaceous crowder BSA and measured by SANS contrast matching in 40% D_2_O. (a) Exemplary SANS data of COR15A without and with 15% v/v of the proteinaceous crowder BSA. The symbols represent the experimental data in log–log representation with solid lines depicting fits using generalized Gauss functions. (b) Radii of gyration (*R*_G_) calculated from Guinier plots and (c) power law scaling coefficients d as derived from the fits in (a) in increasing concentrations of BSA. Error bars depict SD from three measurements. The full set of data in included in Figures S1–S3.

We previously provided *in vitro* evidence for the self‐assembly of COR15A in simple water‐glycerol solutions of increasing osmolarity using different scattering techniques (Shou et al., 2019). Here, we explored if COR15A shows a similar structural behavior under crowding conditions by using bovine serum albumin (BSA) as a biophysical model system for the crowded cellular interior. We investigated the conformational reaction of COR15A to increasing concentrations of the proteinaceous crowder BSA using small angle neutron scattering (SANS) *in vitro*. Scattering techniques are per se not selective for specific molecular species, but will report on all scattering particles in solution. We therefore used deuterated COR15A (dCOR15A) and eliminated the contrast between the non‐deuterated crowder molecule BSA and the buffer by SANS contrast matching in 40% D_2_O. This enabled detection of the dCOR15A scattering signal without any contribution from BSA (Figure 1a, Figures S1–3). For IDPs and polymers, small scattering vectors (*q*) represent the Guinier range and higher *q*‐values the power law scaling range, respectively. From the former, the radius of gyration (*R*_G_) (Figure 1b) can be derived. Guinier and power law region together can be analyzed by the generalized Gauss‐function (Hammouda, 2010), yielding the power law scaling coefficient d (Figure 1c), which reports on local protein chain‐chain interactions, and is as such a proxy for protein compaction. With an increase of BSA up to a volume fraction of almost 25%, which corresponds to 325 g/L, we covered a protein concentration range, which is relevant in a cellular context (Theillet et al., 2014). Increasing the concentration of BSA changes the conformational ensemble of dCOR15A, as expressed in an increase in dimension (*R*_G_) and compaction (*d*) above 8% v/v BSA. Observed crowding effects starting at BSA volume fractions of 8% are, hence, in agreement with the biologically relevant lower limit for eukaryotic cells (Theillet et al., 2014). We previously reported similar changes at high solution osmolarity accompanying COR15A self‐assembly (Shou et al., 2019).

**FIGURE 2:**
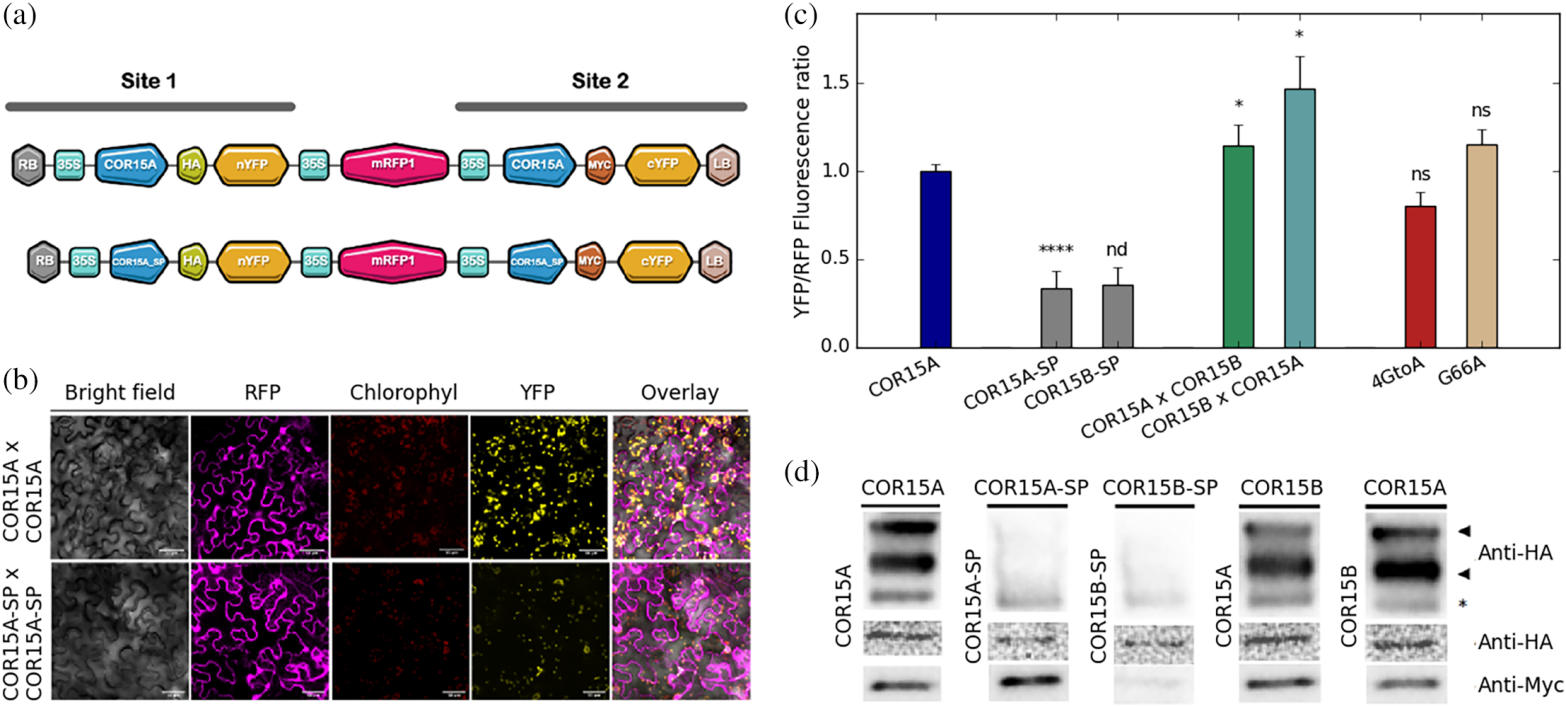
COR15A is associated in oligomeric complexes *in planta*. (a) Visual maps of exemplary rBiFC expression cassettes carrying the full‐length COR15A gene sequence in expression sites 1 and 2, and for a negative control, in which both sites encode the chloroplast signal peptide of COR15A (COR15A‐SP). (b) Representative confocal images of transiently transfected tobacco leaves expressing rBiFC constructs from (a) (Chl, Chlorophyll). (c) Quantification of normalized mean fluorescence from confocal imaging of rBiFC constructs reporting on COR15A homo‐ and COR15A/COR15B hetero‐assembly. We included two COR15A mutants with increased helicity (4GtoA and G66A) and in addition, prepared COR15A‐SP and COR15B‐SP as negative controls. Significant differences to COR15A calculated by unpaired *t*‐test or one‐way ANOVA are indicated (****p ≤ 0.0001, *p ≤ 0.01, ns = not significant, nd = not determined). (d) The upper panels show eluates after Co‐IP, which were resolved by SDS‐PAGE, and immunodetection was performed using *n* = 2 independent biological replicates. Vertical labels correspond to the gene sequence inserted in site 1 and horizontal ones to the one inserted in site 2. The asterisk (*) indicates free reconstituted YFP (28 kDa). Black arrow heads indicate dimeric (~65 kDa) and oligomeric COR15A and COR15A/B complexes (>170 kDa). Lower panels in (d) depict expression controls of both putative interaction partners in the raw protein lysates immunodetected with anti‐HA (upper) and anti‐Myc (lower) antibody. The respective controls for 4GtoA and G66A are shown in Figure S4.

To investigate the physiological relevance of the self‐assembly of COR15A observed *in vitro*, we explored oligomerization of COR15A in tobacco (*Nicotiana benthamiana*) leaf cells by rBiFC and Co‐IP. We employed an expression system facilitating simultaneous expression of two putative interaction partners fused to YFP_n_‐HA or YFP_c_‐Myc, and an intact red fluorescent protein (RFP) for quantification from a single plasmid, ensuring equal gene dosage (Xing et al., 2016; Figure 2a). Confocal microscopy analysis of *N. benthamiana* leaf cells transiently transfected with an expression plasmid carrying two copies of COR15A, one carrying the C‐terminal and one the N‐terminal half of yellow fluorescent protein (YFP) (Figure 2a) resulted in reconstitution of YFP fluorescence, thus revealing dimeric COR15A inside the chloroplast (Figure 2b,c). Co‐immunoprecipitation (Co‐IP) of tobacco leaf lysates transfected with the respective constructs followed by Western Blotting revealed that COR15A forms SDS‐stable complexes of around 65 kDa corresponding to the dimer. We also detected a higher‐order COR15A multimer (Figure 2d). Interaction between the two YFP fragments were excluded using a plasmid carrying two copies of the chloroplast signal peptide of COR15A (COR15A‐SP) in rBiFC and Co‐IP (Figure 2a–d). The signal reduction of this control was not due to degradation, as both fusion proteins were detected by Western Blotting (Figure 2d).

To analyze if COR15A and the closely homologous COR15B hetero‐oligomerize *in planta*, we performed rBiFC and Co‐IP using two constructs with a copy of COR15A and COR15B in sites 1 and 2 (Figure 2a), and vice versa. We detected COR15A/COR15B heterodimers and multimeric complexes comparable to the homo‐oligomers of COR15A in size and abundance (Figure 2c,d). We previously found that substituting conserved glycines with alanines stabilizes transient α‐helicity of COR15A *in vitro*, which ameliorates its stabilizing effect on liposomes during freeze/thawing (Sowemimo et al., 2019). Here, we tested if stabilization of transient α‐helicity *in vitro* translates to oligomerization *in planta*: Two reported COR15A mutants with increased helicity *in vitro*, G66A and 4GtoA, also formed dimers and a higher‐order multimer *in planta* without significant differences compared to COR15A (Figure 2c, Figure S4).

In summary, COR15A forms homo‐ and heterodimers as well as multimeric complexes with its homolog COR15B in the stroma of tobacco leaf cells irrespective of amino acid substitutions that stabilize helicity *in vitro*.

### 2.2 Molecular dynamics simulations reveal a hydrophobic helical core region as the putative COR15A monomer–monomer interaction interface

**FIGURE 3:**
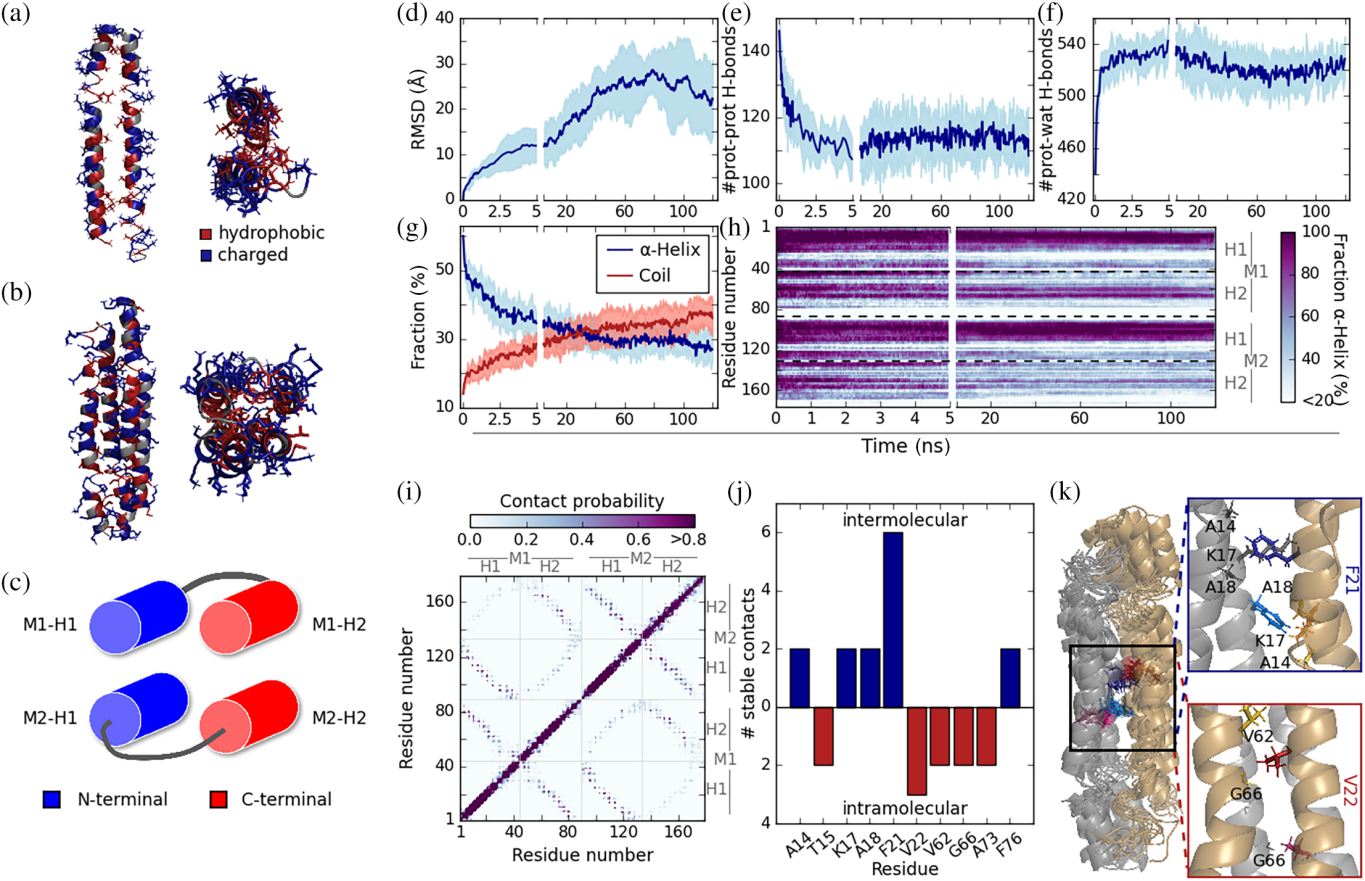
Molecular docking followed by MD simulation of the COR15A dimer reveals intra‐ and intermolecular contacts. Structure predictions of the COR15A monomer (a) and dimer (b). The right panel in (a) and (b) shows the models in a projection along the helix axes. The COR15A monomer model depicts an amphipathic helix–loop–helix structure, with the apolar faces of both helices interacting via hydrophobic residues (a). Molecular docking of the COR15A dimer results in a four‐helix bundle (b). The hydrophobic core is flanked by hydrophilic and charged amino acids. Hydrophobic (red) and charged (blue) residues are highlighted. (c) Illustrative representation of the antiparallel orientation of the two monomers within the dimer with cylindrical shapes referring to the helical regions H1 and H2, and lines to the disordered linkers. The two COR15A monomers in the dimer oppose each other, rotated by 180° such that the loops of the monomers are located on different ends of the dimer. Thus, the N‐terminal helix of the first monomer (M1‐H1) faces the N‐terminal helix of the second monomer (M2‐H1) and vice versa for the C‐terminal helices (M1‐H2 and M2‐H2). (d–i) indicate the stability of the COR15A dimer in terms of changes in RMSD as a measure of the structural distance between backbone atoms (d), intramolecular (e) and protein‐water (f) H‐bonds and secondary structure (g–i) during 120 ns MD simulations. Error bars depict the standard deviation (SD) of 10 simulation replicates. (h) shows unraveling of helical structure resolved per time and residue. Ratio of helicity is represented by color gradient and the first monomer (M1) comprises residues 1 to 89 and second monomer (M2) residues 90–178. (i) Contact maps of the COR15A dimer during MD simulation. Values on the abscissa and ordinate of the contact maps represent the residue number of the COR15A dimer. Contact probabilities were calculated from 10 simulation replicates. (j) shows the most frequently established contacts, derived from all contacts with a contact probability of ≥0.75 from (i). Contacts between the two monomers in the dimer (intermolecular) are shown in blue and contacts within one monomer (intramolecular) are shown in red. (k) shows a ribbon representation of the COR15A dimers after 120 ns simulations, in which the two residues with the most contacts, F21 and V22, are indicated in shades of blue and red, respectively. The insets depict magnified image sections from (k), with the upper inset showing contact amino acids of F21 and the lower one showing contact amino acids of V22.

To identify the oligomerization interface of the COR15A homo‐dimer, we first modeled the monomer, obtaining a helix (H1)‐loop–helix (H2) structure (Figure 3a) similar to previous models (Navarro‐Retamal et al., 2016) and in line with NMR data (Sowemimo et al., 2019). Prediction of the COR15A dimer by molecular docking of monomers yielded a four‐helix bundle (Figure 3b) in antiparallel orientation (Figure 3c). In both predicted models, the hydrophobic surfaces of the amphipathic α‐helices faced each other, forming a hydrophobic core surrounded by polar amino acids (Figure 3a,b). To identify the most essential core residues for oligomerization, we subjected the COR15A dimer to molecular dynamics (MD) simulations at full hydration (Figure 3d–h). This allowed us to study the stability of the COR15A dimer under solvent conditions that destabilize the folded state and thus also the dimer. Similar to the monomer (Figure S5 (Navarro‐Retamal et al., 2016)), the dimer quickly unfolded: the root mean square deviation (RMSD) as a measure of the structural distance between backbone atoms increased (Figure 3d), H‐bonds converted from protein–protein to protein–water (Figure 3e,f), α‐helicity decreased, and random coil fraction increased (Figure 3g). While the H1 helices were stable throughout the simulations, H2 largely unraveled, starting from the terminal regions, resulting in a core with residual helicity (Figure 3h). Contacts among the central regions of H1 and H2 were stable throughout the simulations (Figure 3i,j, Figure S6) and indicated interaction between the two α‐helical cores within one monomer as well as the two monomeric subunits of the dimer. Specifically, two hydrophobic residues, F21 and V22, showed high involvement in these interactions with F21 preferring to establish intermonomer and V22 intramonomer contacts (Figure 3k, Figure S6).

To summarize, MD simulations of the COR15A dimer in solvent conditions destabilizing folding and self‐assembly revealed that interactions within COR15A monomer and dimer are mainly established by two hydrophobic residues F21 and V22.

### 2.3 Mutation of COR15A contact sites hinders self‐assembly *in vitro* and *in planta*

**FIGURE 4:**
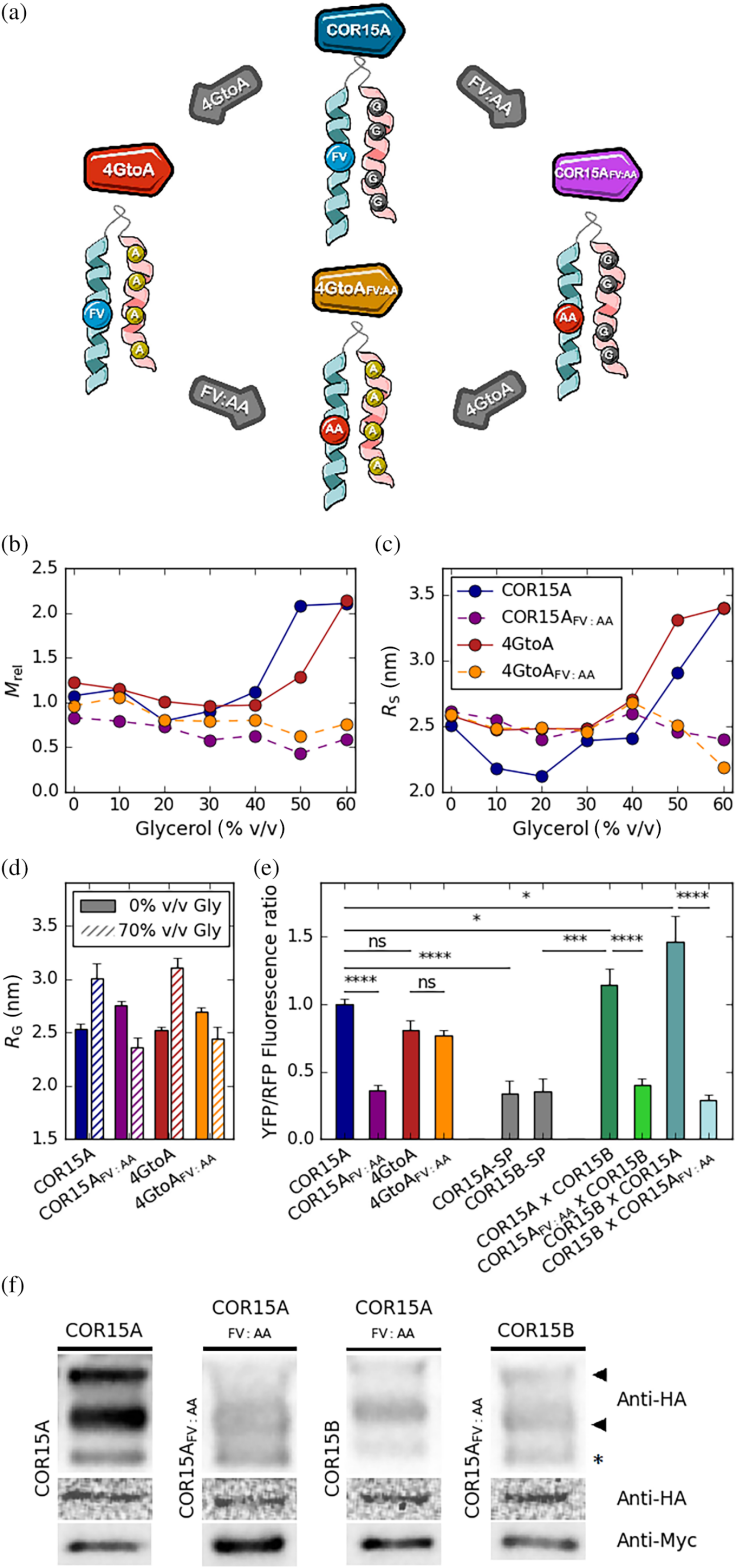
FV:AA mutation suppresses oligomerization *in vitro* and *in planta*. (a) Schematic representation of COR15A WT and mutants investigated in this study (sequence details are provided in Figure S7). The FV:AA substitution is indicated by blue and red spheres and the 4GtoA substitution (referring to the substitution of glycine at positions 52, 66, 79, and 82 with alanine) by gray and yellow spheres. The two helical domains H1 and H2 are schematically depicted in light blue and light red, respectively. Apparent relative masses (*M*_rel_ = *M*/*M*_monomer_) (b) and hydrodynamic radii *R*_S_ (c) of COR15A and its mutants were measured as a function of protein concentration by simultaneous SLS/ DLS experiments and extrapolated to infinite dilution in order to eliminate the influence of intermolecular repulsion or attraction. The resulting *M*_rel_ and *R*_S_ are plotted as a function of glycerol concentration. (d) The radius of gyration (*R*_G_) calculated from SAXS in buffer (filled bars) and in 70% glycerol (shaded bars). Error bars depict SD from at least three measurements. Data on COR15A in (b–d) were taken from (Shou et al., 2019). rBiFC (e) and Co‐IP (f) of COR15A_FV:AA_ and 4GtoA_FV:AA_ to address homo‐oligomerization and COR15A_FV:AA_/COR15B and the swapped construct COR15B/COR15A_FV:AA_ to address hetero‐oligomerization in transiently transformed tobacco leaves. For controls as introduced earlier, compare Figure 2. In (e), asterisks indicate significant differences calculated using one‐way ANOVA statistical analysis (****p ≤ 0.0001, ***0.0001 ≤ *p* ≤ 0.001, *p ≤ 0.5, ns = not significant). The asterisk in (f) represents free reconstituted YFP. In (f), the anti‐Myc antibody was used to pull‐down protein complexes, which were immunodetected using an anti‐HA antibody. Vertical labels correspond to the gene sequence inserted in site 1 and horizontal ones to the one inserted in site 2. Black arrow heads indicate dimeric (65 kDa) and oligomeric COR15A and COR15A/COR15B complexes (>170 kDa).

To probe the importance of F21 and V22 for dimerization, we substituted both residues with alanine in COR15A, yielding COR15A_FV:AA_. In order to study whether and how the stability of secondary structure guides self‐assembly of COR15A *in vitro*, we introduced similar amino acid substitutions in the more helix‐prone COR15A 4GtoA mutant, yielding the 4GtoA_FV:AA_ double mutant (Figure 4a, Figure S7). We investigated recombinant COR15A variants *in vitro* by simultaneous static and dynamic light scattering as a function of water availability, modeled by increasing osmolarities (Figure 4b,c) and by X‐ray scattering at high osmolarity (Figure 4d, Figure S8). While static light scattering provides a direct measure of the molecular mass and thus the average association state (*M*_rel_) of a protein, dynamic light scattering and X‐ray scattering yield information on protein dimensions in terms of the hydrodynamic radius (*R*_S_), and the *R*_G_, respectively. Given the investigated proteins are monomeric, also information on protein compactness can be deduced. We previously used a similar approach to describe the self‐assembly of COR15A (Shou et al., 2019), which showed an increase in *M*_rel_ and dimension at high osmolarity (included in Figure 4b–d as reprinted from (Shou et al., 2019)). Similar to COR15A, 4GtoA responded to increasing osmolarity with an increase in *M*_rel_ and dimension: stabilization of α‐helical structure did thus not disturb the monomer–dimer transition *in vitro*. Strikingly, the FV:AA mutation completely inhibited self‐assembly in response to high solution osmolarity in COR15A_FV:AA_ and also in the double mutant 4GtoA_FV:AA_, as expressed in the lack of an increase in *M*_rel_ and dimension at high osmolarity. The *R*_G_ and *R*_S_ of both variants show a slight decrease at high osmolarity compared to the fully hydrated state. The monomeric nature of COR15A_FV:AA_ and 4GtoA_FV:AA_ allows to contribute this to increasing compaction. We previously reported such a compaction of COR15A with increasing osmolarity: In the absence of glycerol, transformation of the scattering vector *q* according to Kratky showed a progressive increase of the X‐ray scattering intensity with increasing *qR*_G_, as expected for disordered protein chains. At high osmolarity, the Kratky plot showed a distinct peak slightly above *qR*_G_ = 1.73, indicating a certain degree of compaction, although less than expected of a globular protein (Shou et al., 2019). We found a similar behavior for all three COR15A mutants (Figure S8G–I), indicating that protein compaction at high osmolarity occurs in the monomeric and the dimeric COR15A variants and is thus independent of self‐assembly.

Given the simplicity of our *in vitro* solution system, we were interested if the FV:AA mutation would also affect self‐assembly of COR15A in the complex cellular environment. Introduction of the FV:AA substitution in COR15A led to a dramatic suppression of oligomerization in tobacco leaf chloroplasts, as addressed by rBiFC constructs reporting on homo‐oligomerization of COR15A_FV:AA_ and its hetero‐oligomerization with COR15B (Figure 4e). These constructs yielded fluorescence intensity ratios significantly lower than COR15A and COR15A/COR15B, and as low as the negative controls. Similarly, Co‐IP showed a drastic reduction of signals reporting on dimers or higher oligomers (Figure 4f). Interestingly, the double mutant 4GtoA_FV:AA_, similar to 4GtoA, did not yield such a fluorescence reduction (Figure 4e) and thus self‐assembles in the cell. This indicates that the complexity of the cellular environment does not further stabilize the COR15A oligomer compared to the simple *in vitro* environment. It is striking that such a stabilization can be achieved by a minor amino acid substitution, in this case the 4GtoA mutation, which impressively underlines that COR15A self‐assembly in the cell is a highly balanced system. If this stabilization *in planta* is driven either entropically via crowding or enthalpically via the interaction with cellular macromolecules and the role of secondary structure stability, will be exciting objectives to be addressed by future research.

To recapitulate, substitution of the contact residues F21 and V22 prevents self‐assembly, but does not impact compactness of COR15A and of the more helical 4GtoA *in vitro*. The suitability of the simple glycerol‐water *in vitro* model in the study of COR15A self‐assembly is underlined by the finding that FV:AA substitution prevents self‐assembly of COR15A, but not of 4GtoA *in planta*, which is due to entropic or enthalpic contributions of the complex cellular environment.

### 2.4 Destabilization of intramolecular helix–helix interaction perturbs the coil–helix equilibrium of COR15A *in vitro*

**FIGURE 5:**
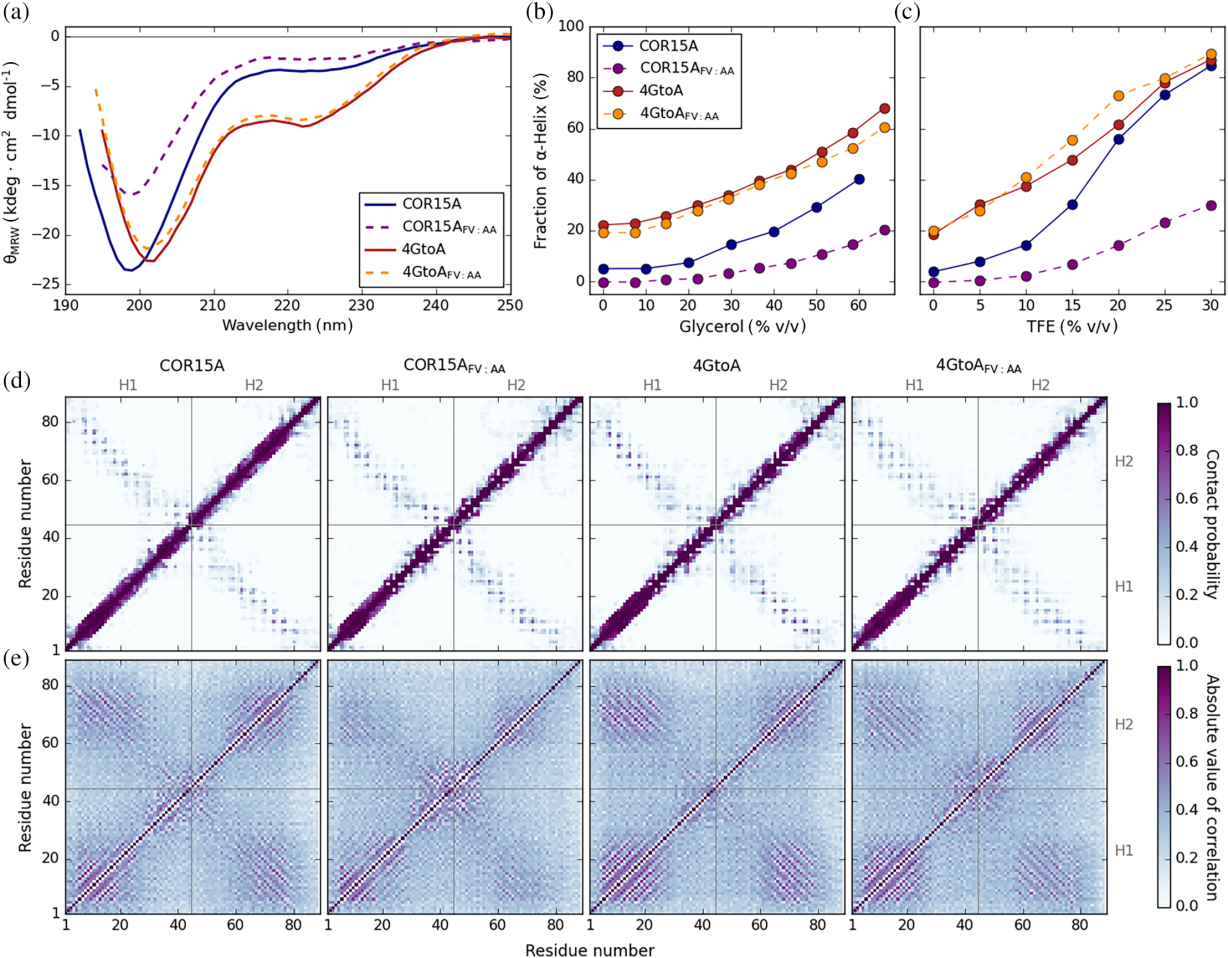
Impact of COR15A mutations on overall disorder, coil–helix transition, intermolecular contacts and structural rigidity. Far‐UV CD spectra of COR15A and its mutants were measured in the fully hydrated state (a) and with increasing concentrations of the cosolutes glycerol and TFE (Figure S9). Fraction of α‐helicity, estimated from the *θ*_MRW_ at 222 nm, of the four proteins as a function of glycerol and TFE concentration are show in (b) and (c), respectively. The datasets for COR15A and 4GtoA in the presence of TFE and for COR15A in the presence of glycerol have been published previously (Sowemimo et al., 2019). Contact maps (d) and correlation of Cα carbons (e) during 120 ns MD simulation of the monomers of COR15A and its mutants. Values on the abscissa and ordinate of the contact maps represent the residue numbers. Contact probabilities and correlations were calculated from 10 simulation replicates for each protein.

Oligomerization of COR15A is accompanied by transition from a disordered state at full hydration to an increasingly α‐helical ensemble at increasing osmolarity *in vitro*; helicity is stabilized in 4GtoA (Sowemimo et al., 2019). We used far‐UV circular dichroism (CD) spectroscopy to investigate the impact of the FV:AA substitution on coil–helix equilibria at increasing concentrations of glycerol and 2,2,2‐trifluoroethanol (TFE) (Figure 5a–c, Figure S9). TFE and osmolytes such as glycerol have different effects on protein structure. Glycerol, next to its effect on water activity, is preferentially excluded from the protein backbone (Timasheff, 2002). While the contact between glycerol and protein is unfavorable per se, it is more unfavorable when the protein is in an unfolded state and this finally results in the stabilization of protein structure, shifting the native protein ensemble to a more compact state (Vagenende et al., 2009). TFE, by contrast, stabilizes α‐helical conformations of proteins by preferential solvation, stabilizing intramolecular H‐bonds (Buck, 1998), which will reveal the maximum propensity of a given protein to form α‐helical structure (Haymet et al., 1999) irrelevant of the physiological relevance. The spectral shapes indicated that all COR15A variants are largely disordered in buffer (Figure 5a). In COR15A_FV:AA_, the overall helicity at increasing concentrations of both cosolvents (Figure 5b,c) was drastically reduced compared to COR15A, reaching about 60% less helical structure at the highest cosolvent concentrations. By contrast, coil–helix transitions in the double mutant 4GtoA_FV:AA_ were similar to the one in 4GtoA. A potential explanation is the stabilization of the folded ensemble due to H1–H2 interaction within the COR15A monomer unit (illustrated in Figure 3c). The FV:AA substitution in COR15A potentially destabilizes this interaction, while the G to A substitutions in 4GtoA likely establish a new intra‐monomeric H1–H2 interaction interface. Thus, the FV:AA substitution does not impact the coil–helix equilibrium of the double mutant 4GtoA_FV:AA_, but only its oligomerization state. To test this hypothesis, we probed H1–H2 interactions within the monomeric subunits of all COR15A variants by MD simulations (Figure S5 and Figure 5d,e), assuming that differences in this interaction would affect ensemble average (contact probabilities, helicity) or dynamic (orientational correlation) structural parameters. The contact probabilities and helicities during 120 ns MD simulations were similar among all COR15A variants (Figure 5d, Figure S5P–T), arguing that ensemble averaged structural parameters were not suited to detect small differences in H1–H2 interaction. The orientational correlation between residues in the protein backbone (Figure 5e) reports on protein dynamics: high correlation indicates low conformational flexibility, arguing for interaction, and correlation of or close to zero implies random movement. The 4GtoA mutation resulted in higher rigidity of H1 although it is situated in the sequence domain which constitutes H2. In COR15A_FV:AA_, the correlation between H1 and H2 was lower than in COR15A, indicating higher flexibility and lower inter–helical interaction. Also, amino acids within H2 were less rigid in COR15A_FV:AA_, indicating that H2 was destabilized although the mutation site was located in H1. In the double mutant 4GtoA_FV:AA_, amino acids of H2 were less impacted by the FV:AA substitution and the correlation between H1 and H2 was higher than in COR15A_FV:AA_. Rigidity of H1 in 4GtoA_FV:AA_ was similarly high as in 4GtoA, indicating that the contacts between H1 and H2 were established in the double mutant 4GtoA_FV:AA_, but not in COR15A_FV:AA_. Thus, the simulations agreed with our hypothesis that intra‐monomeric H1–H2 interactions stabilize the transient α‐helicity of COR15A.

To summarize, substitution of the contact residues F21 and V22 with alanines negatively affects the coil–helix transition of COR15A and this defect is rescued by the 4GtoA substitution. This can be explained by intramonomeric interactions between H1 and H2 that stabilize helicity as indicated by MD simulation.

### 2.5 The FV:AA mutation inhibits functionality of COR15A *in planta* and its functional impact *in vitro* is multifaceted

**FIGURE 6:**
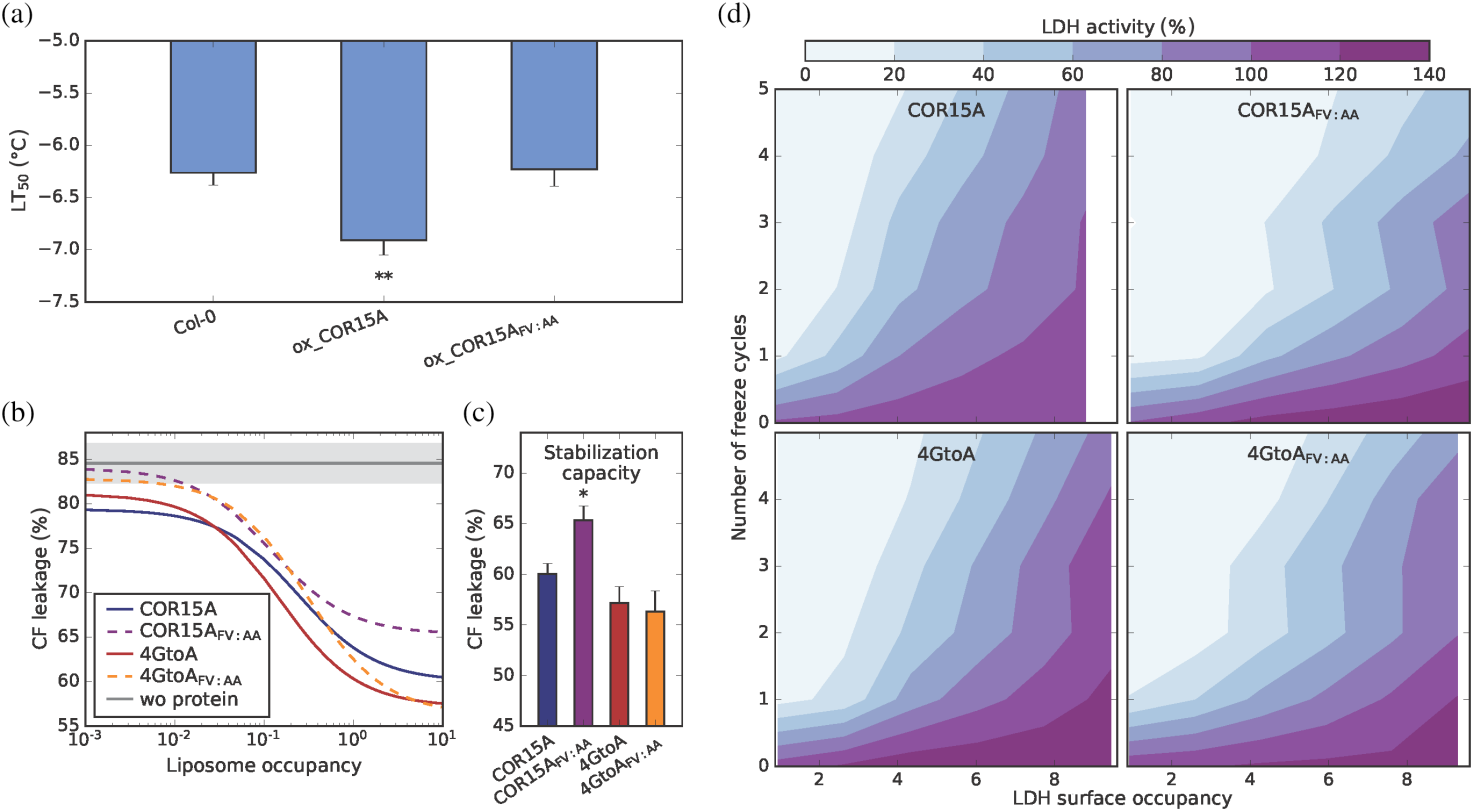
Impact of mutations on COR15A functionality *in vivo* and *in vitro*. (a) The freezing tolerance of not cold acclimated Arabidopsis wild type (Col‐0) and COR15A overexpression lines. Ox_COR15A refers to plants overexpressing COR15A and ox_COR15A_FV:AA_ to plants overexpressing COR15A_FV:AA_. COR15A expression levels of all plant lines are summarized in Figure S10. Freezing tolerance was determined from electrolyte leakage measurements and is indicated as LT_50_. Error bars represent SEM from at least five biological replicates. Asterisks indicate the *p*‐value determined by unpaired Student's *t*‐test <0.01. (b–d) refer to the stabilization of biological targets by COR15A during freeze–thawing *in vitro*. (b) and (c) CF leakage from ICMM liposomes after a freeze–thaw cycle to −20°C. ICMMs were frozen in increasing concentrations of COR15A and its mutants at 12 different liposome surface occupancies. Raw data (Figure S11) were fitted to a dose response model yielding the minimum CF leakage at high liposome surface occupancies as an adaptable parameter. The regression curves are plotted as a function of ICMM surface occupancy in order to account for size differences between the COR15A variants (b). The horizontal bold line shows CF leakage of ICMMs frozen in the absence of (wo) protein ± SEM (gray shaded area). (c) Minimum CF‐leakage values at full surface occupancy derived from nonlinear regression analysis expressing the liposome stabilization capacity with error bars referring to ± SEM and asterisks indicate the *p*‐value determined by unpaired student's *t*‐test <0.05. (d) The effect of COR15A and its mutants on the activity of LDH after zero to five freeze–thaw cycles in liquid nitrogen as a function of LDH surface occupancy. LDH activity is plotted normalized to unfrozen controls in the absence of any protectant.

To evaluate whether suppression of oligomerization affects the *in vivo* function of COR15A (Artus et al., 1996; Thalhammer et al., 2014), we determined the freezing tolerance of Arabidopsis rosettes as LT_50_ (Figure 6a). In the wild type control Col‐0, the expression of COR15A is not induced under not cold acclimated conditions (Figure S10). Overexpression of COR15A led to a significantly increased freezing tolerance of not cold acclimated leaves as previously published (Thalhammer et al., 2014). By contrast, overexpression of COR15A_FV:AA_ yielded an LT_50_ similar to the control. Apparently, the FV:AA substitution led to a loss of function *in planta*.

To dissect how the impact of the diverse amino acid substitutions on protein conformation translates to functional aspects, we assessed the capacity of increasing concentrations of recombinant COR15A variants to stabilize biological targets during freeze–thawing *in vitro* (Figure 6b–d). This concentration dependence is expressed as target surface occupancy, which is a measure for the relative surface area of the target molecule that is theoretically occupied by the COR15A variant at a given protein to target ratio. We use the surface occupancy instead of the more commonly used molecular ratio of protein to target molecules, as it takes into account both molecular mass and size of the COR15A variants. We first addressed the capacity of COR15A variants to stabilize liposomes modeling the lipid composition of inner chloroplast membranes (ICMMs: 40% MGDG, 30% DGDG, 15% SQDG, and 15% EPG) (Webb & Green, 1991) during a freeze–thaw cycle. The fluorescent dye carboxyfluorescein (CF) was incorporated in the ICMMs in a self‐quenching concentration. Upon leakage into the surrounding medium, the dye is diluted and starts to emit fluorescence, which is proportional to liposome damage. Figure 6b shows nonlinear fits of CF leakage data (Figure S11) to a dose–response model. The presence of COR15A resulted in a reduction of CF leakage from ICMMs, corresponding to increasing ICMM stability in a dose‐responsive manner, reaching a plateau at liposome surface occupancies above 1, corresponding to excess protein over liposome, in line with our previous reports (Bremer, Wolff, et al., 2017; Knox‐Brown et al., 2020; Thalhammer et al., 2014). While a similar concentration dependent behavior was observed for all variants (Figure 6b, Figure S11), we found differences regarding stabilization capacity at high occupancies, which were derived from the nonlinear fits and are shown in Figure 6c. These confirm the previously reported higher capacity of the more helical 4GtoA for liposome stabilization compared to COR15A (Sowemimo et al., 2019). Interestingly, COR15A_FV:AA_ had less capacity for liposome stabilization than COR15A, indicating a loss of function going along with the loss of folding and oligomerization, in line with our *in vivo* findings. By contrast, the FV:AA substitution did neither affect stabilization capacity nor helicity in the double mutant 4GtoA_FV:AA_; both are similar to the one of 4GtoA. This indicates that membrane stabilization is exclusively driven by the quantity of amphipathic α‐helices, and not by the oligomeric state.

We previously showed that COR15A stabilizes chloroplast membranes but not a set of target enzymes during freezing *in vivo*. Interestingly, we also found that these enzymes did not aggregate during the chosen freezing treatment, thus not representing suitable targets to address a potential function of COR15A in molecular shielding *in vivo* (Thalhammer et al., 2014). Therefore, our previous results do not rule out the possibility that COR15A does not only stabilize membranes but in addition functions in molecular shielding of target proteins *in vivo*, an open question which cannot be addressed to date due to the lack of knowledge of *in vivo* interaction partners. However, like many other LEA proteins, COR15A can cryoprotect isolated lactate dehydrogenase (LDH) *in vitro*, which was previously assessed by enzyme activity measurements (Lin & Thomashow, 1992; Nakayama et al., 2007; Thalhammer et al., 2014). We first validated that this cryoprotection is reached by the prevention of LDH aggregation, which we assessed by measuring light scattering intensities of LDH subjected to zero to five freeze–thaw cycles in liquid nitrogen alone, in the presence of the four COR15A variants and of the control protein RNase A (Figure S12). We found that LDH alone shows a substantial increase in light scattering intensity, reporting on the formation of aggregates, already after the first freeze–thaw cycle, while all COR15A variants, but not RNase A drastically suppressed aggregation during several freeze–thaw cycles. Having thus shown that cryoprotection of COR15A and its variants is established by molecular shielding, we compared the molecular shielding capacity and effectivity between the COR15A variants. We measured LDH activity after one to five freeze–thaw cycles in liquid nitrogen alone and with all COR15A variants at seven LDH surface occupancies. All variants stabilized the enzyme already at ambient temperature, expressed as considerable increase of residual LDH activity above the one without protectant (Figure 6d). LDH activity decreased with increasing number of freeze–thaw cycles, and all COR15A variants retained LDH activity in a dose‐responsive manner, indicating that none of the mutations affected LDH stabilization capacity. The effectivity of the COR15A variants in LDH stabilization is qualitatively expressed in the shift of the color gradient in both dimensions (number of freeze–thaw cycles and LDH surface occupancies) in Figure 6d. Interestingly, the effectivity of COR15A_FV:AA_ and 4GtoA_FV:AA_, which are both monomeric *in vitro*, was decreased compared to the dimeric COR15A and 4GtoA, indicating that the oligomer is a better enzyme stabilizer than the monomer and that the superior helicity of 4GtoA_FV:AA_ does not lead to superior enzyme stabilization.

In summary, the FV:AA substitution led to a loss of COR15A function *in planta*. *In vitro* experiments allowed to pinpoint the relationship between secondary as well as quaternary structure and *in vitro* multifunctionality. These experiments support the hypothesis that the stabilization of model membranes is regulated by helicity while molecular shielding is driven by the oligomeric state *in vitro*.

## 3 DISCUSSION

Despite ample evidence for the existence of *in vivo* and *in vitro* oligomeric states of LEA proteins (reviewed in (Hernández‐Sánchez et al., 2022)), it is unknown how these shape their intricate structure–function relationship. Our work constitutes a significant step toward elucidating this relationship by combining molecular insights and physiologically meaningful interpretation using an iterative approach between computation, biophysics, biochemistry, and plant physiology. We show that the LEA protein COR15A homo‐ and hetero‐oligomerizes *in planta* and that molecular crowding is a driver of COR15A self‐assembly. We provide molecular details of the inter‐and intra‐molecular interactions facilitating oligomerization of COR15A, and resolve how functional *in vitro* plasticity is encoded in its structural repertoire. We discuss a universal oligomerization mechanism for LEA proteins and its impact on functionality.

**FIGURE 7:**
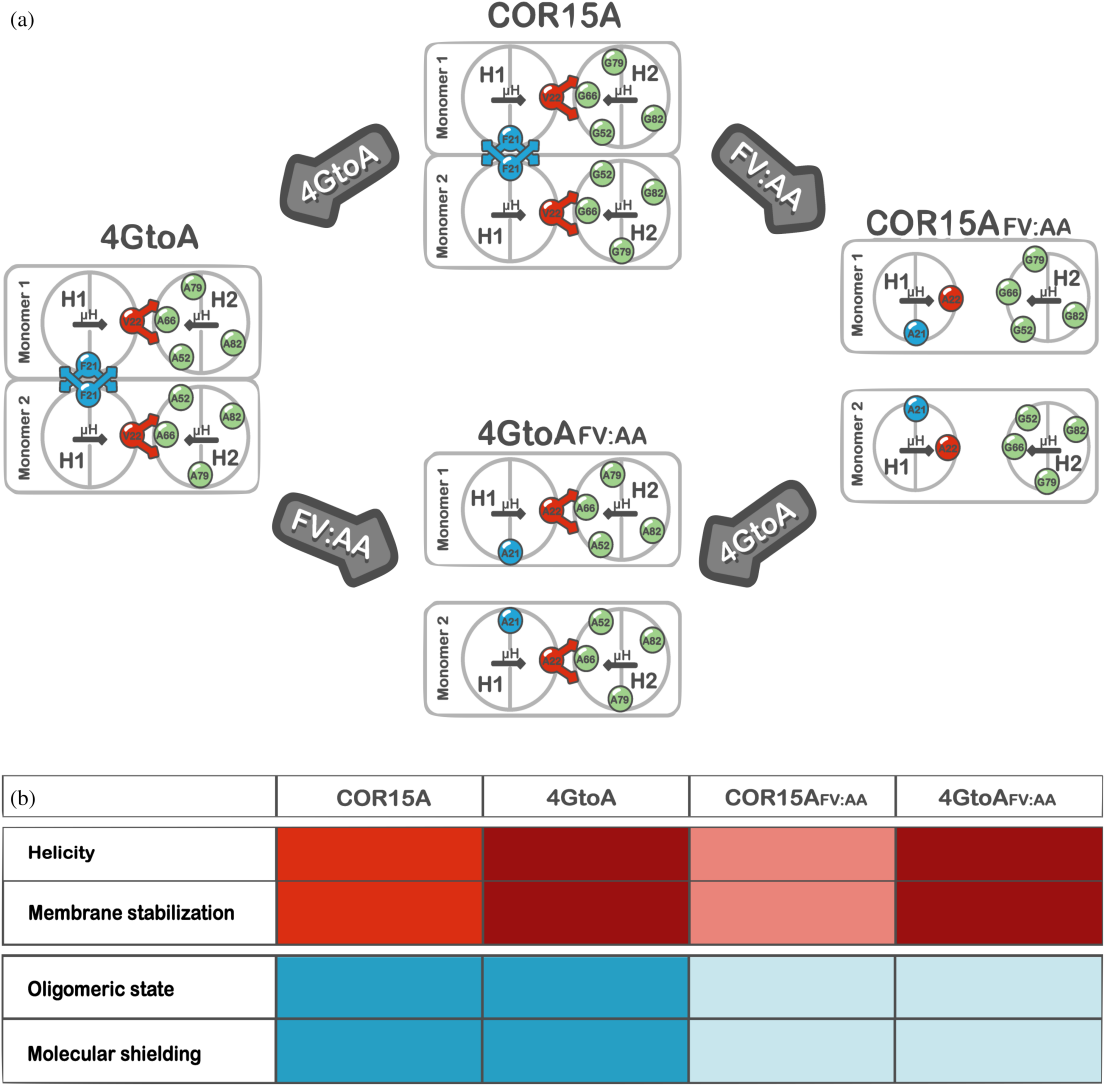
Schematic summary of the impact of COR15A mutations on helicity, oligomeric state and helix–helix interaction of COR15A under conditions promoting folding and their impact on functionality. (a) Protein monomers are represented by gray boxes. H1 and H2 are shown along the helix axes and are depicted as gray circles. Distances between boxes reflect the dimeric or monomeric character of the respective COR15A variant and distances between circles an interaction between H1 and H2. Blue circles indicate amino acid 21, either occupied by an F or an A and red circles amino acid 22, either occupied by a V or an A, both situated on H1. Colored arrows indicate contacts established via these residues. Green circles indicate residues on H2, originally G, but replaced by A in some of the mutants. μH depicts the hydrophobic moment vector calculated from helical wheel projections using the respective amino acid sequences as input (Figure S13) and gray vertical lines indicate the limit between the hydrophobic and the hydrophilic faces of the amphipathic helices. The substitution of four conserved G by A resulted in the oligomeric COR15A variant 4GtoA, characterized by superior α‐helicity *in vitro* (Sowemimo et al., 2019). Substitution of F21 and V22 with alanine in COR15A_FV:AA_ severely destabilized the oligomeric state *in vitro* and *in planta* and largely suppressed the coil–helix transition. In the double mutant 4GtoA_FV:AA_ self‐assembly was suppressed *in vitro*, but not *in planta*, indicating that monomer–monomer interaction was stabilized by the cellular compared to the *in vitro* environment. In addition, the coil–helix transition was not impacted in the double mutant *in vitro*. We rationalized this behavior by interactions between the two transiently α‐helical halves of native COR15A conferring mutual stabilization, as V22, situated on H1, is a major inter‐monomeric contact between H1 and H2. It is oriented in the center of the hydrophobic face, which represents the interface for intramolecular helix–helix interaction and is mainly in contact with V62 and G66. This interaction is destabilized by the V22A substitution in COR15A_FV:AA_, but not in 4GtoA_FV:AA_, where the G66A substitution introduced a more hydrophobic residue and thus rescued H1–H2 interaction and consequently the coil–helix transition. Looking into inter‐monomeric helix–helix interaction, H2 does not represent an interface for self‐assembly. Intermolecular interactions are exclusively driven by H1, primarily by F21, which is oriented perpendicular to μH in the boundary of the hydrophobic face and establishes helix–helix interaction with A14, K17, and A18 of H1 of the second monomer and vice versa, thus stabilizing the COR15A and 4GtoA oligomers. Interestingly, suppression of oligomer formation is not a consequence of the reduced folding propensity of COR15A_FV:AA_, as also 4GtoA_FV:AA_ did not self‐assemble *in vitro*, while its coil–helix transition was not impaired. (b) Summary of the impact of the mutations on protein structure and the assessed *in vitro* functions. Colors indicate the interconnection of structure and function. Light, full and dark red colors reflect (i) low, intermediate and high helicity and (ii) low, intermediate and high membrane stabilization capacity of the four COR15A variants and both patterns are similar. Likewise, dark and light blue colors indicate (i) dimeric and monomeric and (ii) high and low molecular shielding efficiency and again, both patterns are similar.

Our study elucidates new details of structural properties and the molecular nature of helix–helix interactions of COR15A *in vitro* and *in silico*, elaborated on in Figure 7. In summary, the central part of H1 is characterized by intra‐ and inter‐molecular interactions involving coil–helix transitions, matching the definition of an α‐helical molecular recognition feature (α‐MoRF) (Mohan et al., 2006).

We identified two hydrophobic residues, F21 and V22, as the interaction core of this α‐MoRF. Substitution of these residues with alanines led to a loss of self‐assembly and function *in planta*. Unfortunately, conformational aspects on the level of secondary structure can technically not be addressed *in planta*, which is a major drawback on the way to establish how IDP function(s) relate to the modulation of their structural ensembles by the cellular environment per se and by changes in this environment that occur for example upon dehydration, temperature changes, and so on. As such, we can only speculate about the coil–helix equilibrium of COR15A and its relation to oligomerization *in planta*. While interactions of IDPs *in vitro* have been reported to happen in an unfolded state, this special case was found to be possible due to the high opposite net charge of both binding partners (Borgia et al., 2018). In contrast, COR15A, as most LEA proteins, has a net charge of close to zero at neutral pH. Thus, it seems reasonable that oligomerization of COR15A relies on the presence of a hydrophobic surface generated by folding into amphipathic α‐helices. However, it is possible that changes in the cellular solution environment will trigger a rather complex conformational response of LEA proteins that is potentially also modulated by the availability of interaction partners. To bypass these limitations, we addressed the conformational repertoire of COR15A at the level of secondary and quaternary structure *in vitro* by probing a wide range of osmolarities and found that the mutation of the interaction core region impacted both, transient α‐helicity and self‐assembly of COR15A.

Functional *in vivo* studies of LEA proteins are hampered by the identification of molecular targets and suitable experimental conditions. Nevertheless, it has been hypothesized that LEA proteins might carry out multiple functions related to the stabilization of biological structures, and this ability might even be inherent to one and the same LEA protein (Cuevas‐Velazquez et al., 2016; Janis et al., 2022; Tunnacliffe & Wise, 2007). Our functional *in vitro* data revealed that liposome stabilization during freezing exclusively depended on the stability of transient helicity, but not on the oligomeric state. This underlines the concept of COR15A associating with the membrane surface via the hydrophobic faces of its amphipathic helices (Navarro‐Retamal et al., 2018; Thalhammer & Hincha, 2014). LEA proteins stabilize dehydration or freezing sensitive proteins, often isolated enzymes, during freeze–thawing or dehydration–rehydration via an unspecific low‐affinity interaction termed molecular shielding, where aggregation is sterically prohibited by reducing intermolecular cohesion (Chakrabortee et al., 2012). COR15A is competent for molecular shielding *in vitro*, as exemplified by its ability to retain the activity of isolated LDH during freeze–thawing (Lin & Thomashow, 1992; Nakayama et al., 2007; Thalhammer et al., 2014). Strikingly, molecular shielding by COR15A *in vitro* is apparently driven by its ability to self‐assemble, while helicity plays a minor role, if any at all. Thus, our data are in line with the reported size dependence of enzyme stabilization found for LEA proteins of the dehydrin subfamily (Hughes et al., 2013) and provide evidence for the hypothesis of oligomerization being an efficient way to increase the functional capacity of LEA proteins (Hernández‐Sánchez et al., 2014). While our study is the first to report this directly, literature provides hints corroborating the generalization of such a mechanism also for other LEA proteins. For example, a series of studies assign hydrophobic surfaces, often generated by a coil–helix transition, of dehydrin sequences a central role in the stabilization of LDH during freezing (Hara et al., 2017; Ohkubo et al., 2020; Yokoyama et al., 2020). As these studies did not investigate self‐assembly in an oligomerization‐promoting environment, it remained unknown whether LDH stabilization was realized via interaction with exposed hydrophobic surfaces of the target molecules, according to the entropy transfer model (Kovacs et al., 2008), or via hydrophobic interactions leading to self‐assembly, representing classical molecular shielding.

Basic and acidic dehydrins form homo‐oligomers, and also hetero‐oligomerization between a basic and an acidic dehydrin was reported, rendering electrostatics as driving force for oligomerization unlikely (Hernández‐Sánchez et al., 2017). Dehydrins are characterized by different combinations of several conserved short segments (Smith & Graether, 2022) and two of these were found involved in self‐assembly (Hernández‐Sánchez et al., 2017; Upadhyaya et al., 2021). Similarly, an 11‐mer motif conserved in many LEA proteins, but absent in COR15A (Knox‐Brown et al., 2020), was suggested as a main player for self‐assembly (Dure, 1993). Integrating these reports with our data, it is tempting to speculate about a universal mechanism for the homo‐and hetero‐oligomerization of LEA proteins with the unifying characteristic being the existence of an α‐MoRF in the form of a hydrophobic surface. All mentioned segments and motifs feature a regular distribution of hydrophobic residues, separated by mostly charged residues with regular alternation of positive and negative charges. The coil–helix transitions toward amphipathic α‐helices reported for many LEA proteins, among those COR15A, undisputedly allow for such a hydrophobic surface.

Self‐assembly via amphipathic α‐helices often yields coiled‐coil structures. While classical coiled‐coils are characterized by a conserved heptad of amino acids conferring a left‐handed twist (Lupas, 1997), also other periodicities could promote coiled‐coil structures. The 11‐mer motif and also a 15‐residue repeat were suggested to promote self‐assembly into noncanonical, slightly right‐handed coiled‐coils (Dure, 1993; Kühnel et al., 2004). Interestingly, COR15A and COR15B have an almost identical segment matching such a proposed 15‐mer, which for COR15A largely constitutes the identified α‐MoRF, providing a sound explanation for both, homo‐ and hetero‐oligomerization. Although right‐handed coiled‐coils have been successfully designed (Sales et al., 2007), they seem rare in naturally occurring proteins (Kühnel et al., 2004; Stetefeld et al., 2000). One of those, the tetrameric tetrabrachion (Kühnel et al., 2004), has a hydrophobic packing that is quite different from canonical left‐handed coiled‐coils with a larger core filled with water molecules. It is interesting to discuss this finding within the conceptual framework of IDPs, for which protein–protein interaction can yield dynamic complexes with rapid association/dissociation kinetics (Fuxreiter & Tompa, 2012; Sottini et al., 2020). The compaction of COR15A at high osmolarity was lower than for globular proteins and independent of the oligomerization state, suggesting a loose packing of the COR15A dimer, in line with the findings on tetrabrachion. While canonical coiled‐coils are exceptionally stable, a loose packing with rapid association/dissociation rates would allow a swift response to changes in the cellular environment, a hypothesis which will need confirmation by future research. As formation of hetero‐oligomers is a common theme in the structural repertoire of LEA proteins (Ginsawaeng et al., 2021; Hernández‐Sánchez et al., 2017), an important step for decoding their complex structure–function relationship will be to understand the factors determining hetero‐assembly and its impact on functionality. Considering the high number of *LEA* genes in many plant species and their complex transcriptional regulation (Hernández‐Sánchez et al., 2022), it is compelling to hypothesize some degree of multivalency for hetero‐oligomerization, which would allow the LEAome of specific cellular compartments to sample a huge conformational space putatively modulated by the solution environment and molecular targets in an easily reversible manner. This would not only provide an incredible richness of functional facets, but also an effective means for functional regulation with respect to molecular shielding, and might even be a step toward explaining the recent finding of some LEA proteins undergoing liquid–liquid phase separation (Belott et al., 2020; Ginsawaeng et al., 2021; Janis et al., 2022), forming proteinaceous coacervates in solution.

## 4 MATERIALS AND METHODS

### 4.1 Molecular modeling, docking and simulations

The mature amino acid sequence of COR15A without the N‐terminal chloroplast transit peptide (AT2G42540.2) was used for molecular modeling utilizing the I‐TASSER server (Roy et al., 2010; Yang et al., 2015) and the resulting model served as template to generate the mutant structures by MODELER 9.25 (Sali & Blundell, 1993). Predicted model accuracy was evaluated by RMSD, template modeling (TM)‐score, structural alignments against known protein structures from PDB, and the I‐TASSER quality parameter C‐score. The online tools RAMPAGE (Lovell et al., 2003) and MolProbity (Davis et al., 2007) were used for the analysis of stereo‐chemical quality. The amphipathic character of α‐helices was characterized in terms of hydrophobic moments using the software package WebMol (Walther, 1997). COR15A dimer models were generated by docking using the HADDOCK server (van Zundert et al., 2016), and the direction and orientation of hydrophobic moments were analyzed with WebMol. The resulting monomer and dimer structures were virtually identical to state‐of‐art AlphaFold predictions (Jumper et al., 2021; Varadi et al., 2022) (Figure S14), which were performed using the web‐based service ColabFold (https://colab.research.google.com/github/sokrypton/ColabFold/blob/main/AlphaFold2.ipynb) (Mirdita et al., 2022).

MD simulations were used to monitor unfolding of COR15A monomer and dimer structures in water using the Gromacs MD engine (Gromacs 2018.4 and 2020.6) (Abraham et al., 2015) and the OPLS‐AA force field (Jorgensen et al., 1996; Jorgensen & Tirado‐Rives, 1988; Kaminski et al., 2001) as previously described (Navarro‐Retamal et al., 2016). Briefly, models of the folded structures were centered in a dodecahedron box with a minimum edge distance of 15 Å. Solvent was added by the Gromacs tool solvate (Abraham et al., 2015), using the TIP4P (Jorgensen et al., 1983) water model. To guarantee net charge neutrality, six sodium ions were added for the monomer and twelve for the dimer by the Gromacs genion algorithm (Abraham et al., 2015). After energy minimization by steepest descent, the protein was solvent‐equilibrated first in NVT followed by NPT for at least 1 ns. COR15A models were position restrained (1000 kJ mol^−1^ nm^−2^) during equilibration to avoid premature unfolding. The temperature T was controlled by the Bussi–Donadio–Parrinello thermostat (*τ*_T_ = 0.1 ps) (Bussi et al., 2007) and the pressure *P* by the (isotropically applied) Parrinello–Rahman barostat (*τ*_P_ = 2.0 ps) (Parrinello & Rahman, 1981). Overall, 10 replicate simulations of 120 ns each were conducted at 300 K and 1 bar, with 2 fs time step and periodic boundary conditions. A 10 Å cut‐off was used for van der Waals interactions, with a force‐switching function of 10 Å; the Coulomb interactions were treated using particle‐mesh Ewald (Essmann et al., 1995) with real‐space cutoff at 10 Å. Simulation replicates had independent initial velocities, and were averaged prior to all downstream analyses. Covalent bonds were constrained to their equilibrium lengths by (fourth‐order single‐iteration) parallel linear constraint solver (P‐LINCS) (Hess et al., 2008) in the proteins and by SETTLE (Miyamoto & Kollman, 1992) in water.

RMSD of backbone atoms was calculated by the Gromacs tool rms and H‐bond formation by hbond (Abraham et al., 2015). The latter used arrangement criteria of distance ≤3.5 Å between donor and acceptor and a bond angle >120°. Contacts between amino acids were monitored over simulation time using a closest‐distance cutoff of 3.5 Å corresponding to the length of an H‐bond and were computed using the mindist function implemented in Gromacs (Abraham et al., 2015). Contact probabilities were calculated by averaging over all snapshots of all trajectories. Secondary structure assignment was done using the Gromacs do_dssp tool based on the DSSP algorithm (Kabsch & Sander, 1983; Touw et al., 2015). Analysis of backbone orientational correlation was done according to (Virtanen et al., 2020). Briefly, the orientational correlation between protein backbone residues *i* and *j* was analyzed by calculating the average dot product ⟨*v*_*i*_·*v*_*j*_⟩, where *v*_*i*_ is the normalized vector between Cα_*i*_ and Cα_*i*+1_, and the average is over the trajectory. A resulting positive or negative value indicates a corresponding orientational correlation. Since we were only interested in whether there was a correlation or not, the absolute values were used. Random orientations of vectors result in an average value zero, as do vectors constantly oriented orthogonal to each other, since the dot product is zero for this case.

### 4.2 Molecular cloning

*COR15A* and *COR15B* (AT2G42530.1) sequences were amplified from *A. thaliana* cDNA. G66A and 4GtoA COR15A variants (Sowemimo et al., 2019) were synthesized using plant codon optimization (GeneScript, Rijswijk, Netherlands). COR15A_FV:AA_ and 4GtoA_FV:AA_ sequences were generated by site‐directed mutagenesis (Karnik et al., 2013) using the respective plasmids carrying *COR15A* and *4GtoA* DNA as templates.

For stable transformation in Arabidopsis, Gateway‐compatible destination vectors were used. Entry constructs were generated in pENTR/D‐TOPO (Thermo Fisher Scientific, Waltham, Massachusetts, USA) and sub‐cloned into the pMDC32 destination vector by LR reaction using LR Clonase II (Thermo Fisher Scientific) according to manufacturer's instructions. Constructs were verified by sequencing using the NOS‐T Rv primer.

To obtain rBiFC and Co‐IP constructs, entry clones for transient transformation of tobacco leaves were generated by PCR amplification of the open reading frames of the *COR15A*, *COR15B*, *G66A*, *4GtoA*, *COR15A*_*FV:AA*_, and *4GtoA*_*FV:AA*_ genes using gene‐specific primers containing Gateway recombination sites for attB3/B2 (site 1) or attB1/B4 (site 2) borders. As a control to exclude false positive signals resulting from natural interactions between the two YFP fragments in the chloroplast, we additionally generated entry clones containing the signal peptide encoding sequences of COR15A and COR15B (AA 1–49). Amplified attB‐products were BP‐cloned into pDONR221‐P3P2 or pDONR221‐P1P4 using BP Clonase (Thermo Fisher Scientific). The resulting entry constructs were sub‐cloned into pBiFCt‐2in1‐CC (Grefen & Blatt, 2012) by LR reactions using LR Clonase II plus (Thermo Fisher Scientific). The identity of all constructs was confirmed by sequencing (LGC Genomics, Berlin, Germany) using HA‐tag Rv and Myc‐tag Rv primers. All cloning primers and generated constructs are summarized in Tables S1 and S2, respectively. All constructs used for rBiFC and/or Co‐IP are additionally listed in Figure S15, including exemplary confocal images.

### 4.3 Plant material and growth conditions

All *A. thaliana* lines used in these studies had the Columbia‐0 (Col‐0) background. For the generation of COR15A_FV:AA_ overexpression lines, seeds were vernalized for 48 h at 4°C and grown in half‐strength agar Murashige and Skoog (MS 1/2) medium supplemented with 1% sucrose. Plates were grown vertically in long day conditions (16 h day length with 120 μmol m^−2^ s^−1^ light intensity at 22°C, 60% relative humidity) for 2 weeks, plants were then transferred to soil and grown in a greenhouse (16 h day length, 21/19°C day/night, 50% relative humidity) until transformation. Transgenic COR15A overexpression lines (ox_COR15A) were described previously (Thalhammer et al., 2014). Plants for electrolyte leakage experiments were grown as described (Zuther et al., 2019) with two weeks growth under short day conditions after pricking for more leaf material. Rosettes for qPCR analysis were harvested and immediately frozen in liquid nitrogen. Expression levels of *COR15A* were assessed by qRT‐PCR as previously described (Thalhammer et al., 2014) and are shown as relative expression values normalized to the average of the two housekeeping genes *actin* and *GAPDH* (2^−ΔCT^) (Figure S10).

*N. benthamiana* plants were grown for 5 weeks in growth chambers at a long‐day photoperiod (16 h day length, 250 μmol m^−2^ s^−1^, 22/18°C day/night) until transformation.

### 4.4 Plant transformation

Transgenic Arabidopsis lines were generated by floral dip (Zhang et al., 2006) using *Agrobacterium tumefaciens* GV3101 carrying pMDC32‐ COR15A_FV:AA_. Transgenic lines were grown in MS 1/2 medium with 1% sucrose and 25 μg/μL hygromycin B until T3 generation to reach homozygosity. Transgene expression levels were analyzed by qRT‐PCR using specific primers (Table S1). Transient transformation in tobacco was carried out using the *A. tumefaciens* strain GV3101 harboring the pBiFCt‐2in1‐CC expression vectors described in Table S2. *A. tumefaciens* cells were grown in Luria Broth (LB) at 28°C with shaking at 200 rpm overnight to an OD_600_ of 2–4. Cells were collected by centrifugation at 1800*g*, resuspended in 3 mL infiltration buffer (10 mM MES‐KOH (pH 5.7)), 10 mM MgCl_2_, 200 μM acetosyringone (3′,5′‐dimethoxy‐4′‐hydroxyacetophenone) and incubated for 4 h at room temperature (RT) under dark conditions. Agrobacterium cells were co‐infiltrated in the abaxial side of 5 weeks old tobacco leaves together with cells from an *A. tumefaciens* GV3101 strain harboring the p19 suppressor of gene silencing, obtained from D. Baulcombe (Department of Plant Science, Cambridge University, UK) (Garabagi et al., 2012) in a 1:1 ratio and kept in dark at 22–23°C for 3 days.

### 4.5 Expression and purification of recombinant proteins in *Escherichia coli*

Recombinant COR15A variants were expressed in *E. coli* BL21(DE3) cells grown in autoinduction medium (Studier, 2005) to an OD_600_ of 1 at 37°C and subsequently at 20°C over night. Cell harvesting, lysis and protein purification was done as described previously (Sowemimo et al., 2019). Protein purity was confirmed by SDS‐PAGE and dynamic light scattering. Concentration of recombinant proteins was determined from UV absorption spectra using the specific absorption (280 nm, 1 cm path length, 1 mg/mL) calculated from the amino acid sequences using the Prot Param tool (Gasteiger et al., 2005). Fully deuterated COR15A (dCOR15A) was produced at the National Deuteration Facility of ANSTO (Australian Nuclear Science and Technology Organization), Lucas Heights, New South Wales, Australia.

### 4.6 Confocal laser scanning microscopy (CLSM) and image processing for rBiFC

Subcellular localization and protein–protein interaction analyses were performed in transiently transformed tobacco leaves by recording the fluorescence of YFP and RFP as described (Grefen & Blatt, 2012). Confocal images were acquired in a Leica TCS SP5 microscope with a 40× water immersion objective (Leica Biosystems, Wetzlar, Germany). YFP, RFP and chlorophyll fluorescence was detected using excitation/emission wavelengths of 512/520–540, 566/600–607, and 488/680–720 nm, respectively. All images were acquired using sequential scanning settings. For rBiFC, YFP and RFP fluorescence signals were collected using 25–35 z‐stacks with 0.8 μm spacing at low resolution to avoid bleaching. Imaging conditions were kept identical for all samples and experiments. Image processing was performed using IMARIS Bitplane Essentials software (Oxford Instruments, Abingdon, UK). YFP fluorescence in the chloroplast was ratioed to the RFP signal (YFP/RFP) per μm^2^. Each experiment was repeated three times under identical conditions. Statistical analyses were performed using 99% confidence intervals, and significant differences based on unpaired *t*‐test and one‐way ANOVA were determined using GraphPad Prism (GraphPad Software, Boston, Massachusetts, USA). Data were plotted as means ± SEM of 20 images randomly selected over the surface of four leaf samples (five images per leaf ).

### 4.7 Co‐IP

Co‐immunoprecipitation experiments were performed using lysates of tobacco leaves transfected with pBiFCt‐2in1‐CC vectors (Table S2) as described in (Kosmacz & Skirycz, 2020) with slight modifications. Briefly, transfected tobacco leaves were frozen in liquid nitrogen and ground to powder using mortar and pestle, which was mixed with lysis buffer (50 mM Tris HCl, pH 7.4, 100 mM potassium acetate, 2 mM magnesium acetate, 5 mM DTT, 1 mM phenylmethylsulfonyl fluoride, 0.1 mM sodium orthovanadate, 1 mM sodium fluoride, cOmplete, EDTA‐free Protease Inhibitor Cocktail (Roche, Basel, Switzerland)), 1 U/μL RNasin plus RNase inhibitor (Promega, Madison, Wisconsin, USA) in a 1:1 (w/v) ratio. Total soluble protein was separated by centrifugation, protein concentration was determined by Bradford assay and adjusted to equal amounts. Protein fractions were kept on ice until processing. 500 μL of 1.2 g/L soluble protein were incubated with 30 μL of Dynabeads protein A (Thermo Fisher Scientific) equilibrated in DEPC‐treated PBS for 15 min under rotation. After separation of the beads by magnetic force, the supernatant was mixed with 4 μg Anti‐Myc tag rabbit IgG antibody (ab9106, Abcam, Cambridge, UK) and rotated for 1 h at RT. The excess antibody was removed by centrifugation and the resultant pellet was resuspended in 500 μL lysis buffer. 60 μL equilibrated beads were added and incubated for 15 min at RT under rotation. The beads were separated from the lysate and washed three times with lysis buffer at RT.

### 4.8 SDS‐PAGE and Western Blot

Western Blotting was used for detection of co‐immunoprecipitated protein and to verify equal expression of fused proteins. Therefore, protein‐loaded beads and raw protein lysates were resolved by SDS‐PAGE according to (Schägger & von Jagow, 1987). Proteins were immunoblotted as described in (Thalhammer et al., 2014) and probed with Anti‐HA tag or Anti‐Myc tag rabbit IgG antibody (ab9110, ab9106, Abcam, Cambridge, UK) as primary and Goat anti‐Rabbit IgG (H + L) Cross‐Adsorbed, HRP (Thermo Fisher Scientific) as secondary antibodies, respectively. Peroxidase activity was detected using the Novex ECL Chemiluminescent Substrate Reagent Kit (Thermo Fisher Scientific) according to the manufacturer's instructions.

### 4.9 CD spectroscopy

Far‐UV CD spectra of all proteins were measured in a Jasco J‐815 or J‐715 spectropolarimeter (Jasco, Pfungstadt, Germany) equipped with Peltier thermostat‐controlled cell holders in quartz cuvettes of 1 mm path length (Hellma, Muellheim, Germany) at 23°C. To guarantee exact concentrations, proteins were diluted from concentrated stock solutions in 20 mM NaH_2_PO_4_/Na_2_HPO_4_, pH 7.4 by weighing into the same solvent containing defined concentrations of glycerol (≥99.5%, Carl Roth GmbH, Karlsruhe, Germany) to increase osmolarity or TFE (≥99%, Carl Roth GmbH, Karlsruhe, Germany), as a stabilizer of α‐helical structure. The instrument was calibrated with 1S‐(+)‐10‐camphorsulphonic acid and α‐helix ratio was estimated using *θ*_MRW_ at 222 nm (Chen et al., 1972).

### 4.10 Combined static and dynamic light scattering (SLS/DLS)

SLS/ DLS measurements of proteins were done in 20 mM NaH_2_PO_4_/Na_2_HPO_4_, pH 7.4 supplemented with 20 mM NaCl for charge screening containing the indicated amounts of glycerol as described previously (Shou et al., 2019). Due to the high viscosity and density of glycerol, all protein solutions were prepared by weighing and were subjected to ultracentrifugation at 60,000*g* for 30 min to remove dust, air bubbles and large protein aggregates. DLS measurements of liposomes were done on freshly prepared liposomes diluted in water. Light scattering of LDH was measured without further dilution of the freeze‐thawed samples and scattering intensities were normalized to toluene. Briefly, simultaneous DLS and SLS experiments were executed in 3 mm‐pathlength micro‐fluorescence cells (105.251‐QS, Hellma, Muellheim, Germany) using a custom‐built apparatus equipped with a high quantum yield avalanche photo diode, a 0.5 W diode‐pumped continuous‐wave laser (Cobolt Samba 532 nm, Cobolt AB, Solna, Sweden), and an ALV 7002‐25 ns correlator (ALV‐GmbH, Langen, Germany) at 23°C at a scattering angle of 90°C. Refractive indices of all solvents were determined using an Abbe refractometer and viscosities using a rolling ball viscometer (AMVn, Anton Paar GmbH, Graz, Oesterreich) at 23°C and are listed in (Shou et al., 2019). Mean scattering intensities from SLS and time‐autocorrelation functions of the fluctuations in the instantaneous scattering intensities from DLS were recorded over 10 s with at least 150 accumulations. Translational diffusion coefficients D were calculated from autocorrelation functions obtained from the DLS measurements using the CONTIN algorithm (Provencher, 1982) and transformed to apparent *R*_S_ using the Stokes–Einstein equation *R*_S_ = *k*_B_ *T*/(6*πηD*), where *k*_B_ is Boltzmann's constant, *T* is the temperature in Kelvin, and *η* is the solvent viscosity. The apparent molecular mass was calculated using the mean scattering intensity from SLS measurements, with toluene as a standard. Refractive index increments (*dn*/*dc*) were calculated based on the amino acid sequence of COR15A and the respective mutants using the SEDFIT software version 16.1c (Schuck, 2000). In each solvent, SLS/DLS data were collected for five to six protein concentrations between 0.5–5 mg/mL. These apparent molecular masses and *R*_S_ were extrapolated to infinite dilution to obtain the hydrodynamic radius and molecular mass of COR15A at each respective solvent condition after elimination of the influence of the remaining intermolecular interactions. Molecular masses *M* are shown as relative masses *M*_rel_ (*M*/*M*_monomer_) for a direct depiction of the average oligomeric state.

### 4.11 Small angle X‐ray scattering

Small angle X‐ray scattering (SAXS) was measured on an in‐house SAXS instrument (GANESHA, Xenocs, Grenoble, France) located at JCNS‐1/IBI‐8. Measured protein concentration by SAXS was 10 mg/mL for all samples. SAXS data were radially averaged using the instrument software and the solvent contribution was subtracted using python scripts. The scattering vector *q* is defined in this work as *q* = 4*π*/λ*sin(*θ*/2) with the incident X‐ray or neutron wavelength λ and the scattering angle *θ*. No radiation damage was found in the SAXS data and $R_{G}$ have been calculated from the initial slope of the measured SAXS intensities $I\left(q\right)$ according to $\ln\left[I\left(q\right)\right]\sim -R_{G}^{2}q^{2}/3$ using the Guinier approximation. Guinier fits remained linear up to $q_{\max}R_{G}\leq 1.3$, where $q_{\max}$ is the maximal *q*‐vector that was included in the fits and fulfills the limiting Guinier criterium. SAXS data have also been fitted using generalized Gauss functions (Arbe et al., 2016; González‐Burgos et al., 2020; Hammouda, 2012) as shown in Figure S8. Fits have been performed using SasView 5.0.6 (https://www.sasview.org) and python scripts.

### 4.12 Small angle neutron scattering

SANS experiments were performed on the extended *Q*‐range small‐angle neutron scattering diffractometer (EQ‐SANS) beamline of the Spallation Neutron Source at the Oak Ridge National Laboratory (Heller et al., 2018). Measured dCOR15A concentration was approximately 4 mg/mL for all samples. Buffer composition was 10 mM NaH_2_PO_4_/Na_2_HPO_4_, pH 7.4, 150 mM NaCl in 40% D_2_O (99.9% atom D, Sigma‐Aldrich, St. Louis, Missouri, USA) to match out the scattering contribution of BSA (Banks et al., 2018). BSA concentrations were measured optically and were in the concentration range from 50 to 320 mg/mL, corresponding to a range of volume fractions of 4–25% v/v, as determined using a partial specific volume of *ν* = 0.735 mL/g (Stadler et al., 2011). Samples were centrifuged at 60,000*g* for 30 min prior to the SANS experiment. Samples were loaded in 1 mm pathlength circular “banjo” Hellma quartz cuvettes and placed in a temperature controlled sample changer set to 25°C. Data were collected at 60 Hz operation mode at two instrument settings: at 2.5 m sample‐to‐detector distance with a wavelength band of 2.5–6.4 Å and at 4m sample‐to‐detector distance with a wavelength band of 6–9.6 Å, covering a combined *Q*‐range of 0.006–0.6 Å^−1^. The measured scattering data were corrected for detector efficiency, scattering from the empty cells and buffer, placed on absolute scale and circularly averaged via the data reduction software drtsans (https://doi.org/10.11578/dc.20220109.1). Guinier fits have been performed to determine $R_{G}$ in the limit $q_{\max}R_{G}\leq 1.1$ (Figure S2). SANS data have also been fitted using generalized Gauss functions (Arbe et al., 2016; González‐Burgos et al., 2020; Hammouda, 2012) as shown in Figure S1.

### 4.13 Estimation of surface occupancies

The surface occupancy is an estimate for the relative surface area of the target structure (liposomes or LDH) theoretically occupied by the respective protectant (COR15A variant), which we use to account for differences in *R*_S_ and molecular mass of the different protectants. We assume a spherical target surface area (*A*_target_ = 4*πR*_S_^2^) and a spherical plane contact area of the protectant and the target structure (*A*_prot_ = *πR*_S_^2^). The *R*_S_ of 4.3 nm for LDH was taken from (Zinkham et al., 1968). To relate the surface occupancy to the ratio between protein in solution and amount of lipids used for liposome preparation, we took into account the total number of lipids per spherical unilamellar liposome (*n*_l/tot_) with $n_{l,\mathrm{tot}}=\sum f_{l*}\frac{\left[4*\pi *{\left(R_{S,\text{lipo}}\right)}^{2}+4*\pi *{\left(R_{S,\text{lipo}}-d\right)}^{2}\right]}{a}$, (Mozafari et al., 2021), where (*f*_*l*_) is the fraction of each respective lipid species in the liposome mixture, *R*_S,lipo_ the *R*_S_ of the liposome (nm), *d* the bilayer thickness (nm), and (*a*) the lipid head group area for the respective lipid (nm^2^), both taken from (Navarro‐Retamal et al., 2018) for monogalactosyldiacylglycerol (MGDG), digalactosyldiacylglycerol (DGDG), and sulfoquinovosyldiacylglycerol (SQDG) and from (Shahane et al., 2019) for egg l‐*α*‐phosphatidylglycerol (EPG). Thus, the surface occupancy can be expressed as $\frac{c_{\text{Prot}}*a_{\text{Prot}}*n_{l,\mathrm{tot}}*{\mathrm{MW}}_{\text{Lipid}}}{a_{\text{target}}*c_{\text{Lipid}}*{\mathrm{MW}}_{\text{Prot}}}$ for liposomes and $\frac{c_{\text{Prot}}*a_{\text{Prot}}*{\mathrm{MW}}_{\mathrm{LDH}}}{a_{\text{target}}*c_{\mathrm{LDH}}*{\mathrm{MW}}_{\text{Prot}}}$ for LDH, where *c*_Prot_, *c*_LDH_, and *c*_Lipid_ are the concentrations (mg/mL) and *MW*_Prot_, *MW*_LDH_, and *MW*_Lipid_ are the molecular mass (g/mol) of the protectant and the targets, respectively.

### 4.14 Formation of liposomes and CF leakage assay

Stability of inner chloroplast membrane mimicking (ICMM) liposomes during a freeze/thaw cycle in the absence and presence of LEA proteins was assessed as previously described (Sowemimo et al., 2019). All lipids were purchased from Avanti Polar Lipids (Alabaster, Alabama, USA) and dissolved in chloroform. 40% MGDG, 30% DGDG, 15% SQDG and 15% EPG were mixed and dried under N_2_ gas at 60°C. Traces of solvent were removed under vacuum overnight or in a speed vac for at least an hour. Lipids were hydrated in 100 mM CF (Molecular Probes, Eugene, Oregon, USA), 10 mM TES, 0.1 mM EDTA, pH 7.4. Liposomes were extruded with a hand held extruder (Avanti Polar Lipids, Alabaster, Alabama, USA) through two polycarbonate membranes with 100 nm pore size (Nucleopore, GE Healthcare, Freiburg, Germany). Liposomes were separated from free CF by size exclusion chromatography, using a S75 13/300 size exclusion column equilibrated and eluted with 10 mM TES, 50 mM NaCl, 0.1 mM EDTA (pH 7.4) connected to an NGC Chromatography system (Bio‐Rad, Hercules, California, USA), utilizing the absorption at 280 nm. Liposomes were mixed with protein solutions (COR15A variants or RNase A from bovine pancreas (Merck KGaA, Darmstadt, Germany) as control) in the same buffer at 12 liposome surface occupancies per protein, resulting in a final lipid concentration of about 0.4 mM. Samples were rapidly frozen in an ethylene glycol bath precooled to −20°C and after 2 h thawed at RT. CF fluorescence was determined with an Infinite M1000 PRO fluorescence plate reader (Tecan, Männedorf, Switzerland) using an excitation wavelength of 492 nm and an emission wavelength of 515 nm before and after disrupting the liposomes with Triton X‐100 (Thermo Fisher Scientific) in 96‐well fluorescence plates in triplicates. COR15A and RNase A were included in each individual experiment to ensure reproducibility among independent experiments and each COR15A variant was probed in at least three independent experiments. CF leakage from the liposomes was calculated as described previously (Hincha et al., 2002), plotted as a function of surface occupancy and fitted by nonlinear regression using a dose–response model: CFleakage=min+max−min1+10^logEC50−x.

### 4.15 LDH activity and aggregation measurements

LDH activity before and after one to five freeze–thaw cycles in liquid nitrogen in the absence or presence of the COR15A variants in seven different surface occupancies was measured as described previously (Thalhammer et al., 2014) using tetrameric LDH from rabbit muscle (Roche, Basel, Switzerland) and averaged over at least six technical replicates. COR15A and BSA (Carl Roth, Karlsruhe, Germany) were included in each individual experiment to ensure reproducibility among independent experiments.

For the aggregation assay, 200 μL 0.15 g/L LDH in 30 mM NaH_2_PO_4_/Na_2_HPO_4_, pH 7.4 alone or in the presence of the COR15A variants or RNase A from bovine pancreas (Merck KGaA, Darmstadt, Germany) as control at surface occupancies of four to five were subjected to zero to five freeze–thaw cycles in liquid nitrogen. Samples were kept in liquid nitrogen for at least 15 min and subsequently thawed at room temperature. Aggregation was assessed via light scattering measurements (see above).

### 4.16 Determination of leaf freezing tolerance

Freezing tolerance of Arabidopsis leaves was determined as LT_50_, defined as the temperature at which the plasma membrane is sufficiently damaged to release 50% of electrolytes as previously published (Thalhammer et al., 2014; Thalhammer et al., 2020). In brief, rosettes from different plants were frozen over a −1 to −20°C temperature range in five replicates each, slowly thawed on ice, immersed in distilled water and shaken for 16 h at 4°C. Electrolyte leakage was determined from conductivity measurements before and after boiling the leaves and normalized to unfrozen controls. Electrolyte leakage was plotted as a function of temperature and fitted by nonlinear regression using a dose–response model: Electrolyte leakage=min+max−min1+10^logEC50−x.

## AUTHOR CONTRIBUTIONS

**Itzell Hernández‐Sánchez**: Investigation; formal analysis; visualization; writing—review and editing; **Tobias Rindfleisch**: Visualization; investigation; formal analysis; writing—review and editing; **Jessica Alpers**: Investigation; writing—review and editing; **Martin Dulle**: Investigation; writing—review and editing; **Christopher J. Garvey**: Supervision; resources; writing—review and editing; **Patrick Knox‐Brown**: Methodology; writing—review and editing; **Markus S. Miettinen**: Supervision; methodology; writing—review and editing; **Gergely Nagy**: Investigation; writing—review and editing; **Julio M. Pusterla**: Investigation; **Agata Rekas**: Investigation; **Keyun Shou**: Investigation; **Andreas M. Stadler**: Investigation; conceptualization; project administration; formal analysis; writing—review and editing; **Dirk Walther**: Resources; supervision; writing—review and editing; **Martin Wolff**: Methodology; software; writing—review and editing; **Ellen Zuther**: Investigation; formal analysis; writing—review and editing; **Anja Thalhammer**: Conceptualization; project administration; investigation; formal analysis; writing—original draft.

## CONFLICT OF INTEREST STATEMENT

The authors declare no conflicts of interest.

## Supporting information


**Data S1.** Supporting Information.
