# Supplementary material for: Functional in vitro diversity of an intrinsically disordered plant protein during freeze–thawing is encoded by its structural plasticity
Source: Protein Sci. 2024 Apr 24;33(5):e4989. doi: 10.1002/pro.4989 (PMC11043620; doi:10.1002/pro.4989)
Supplement: Supplementary file 1 — Data S1. Supporting Information. [file PRO-33-e4989-s001.pdf]

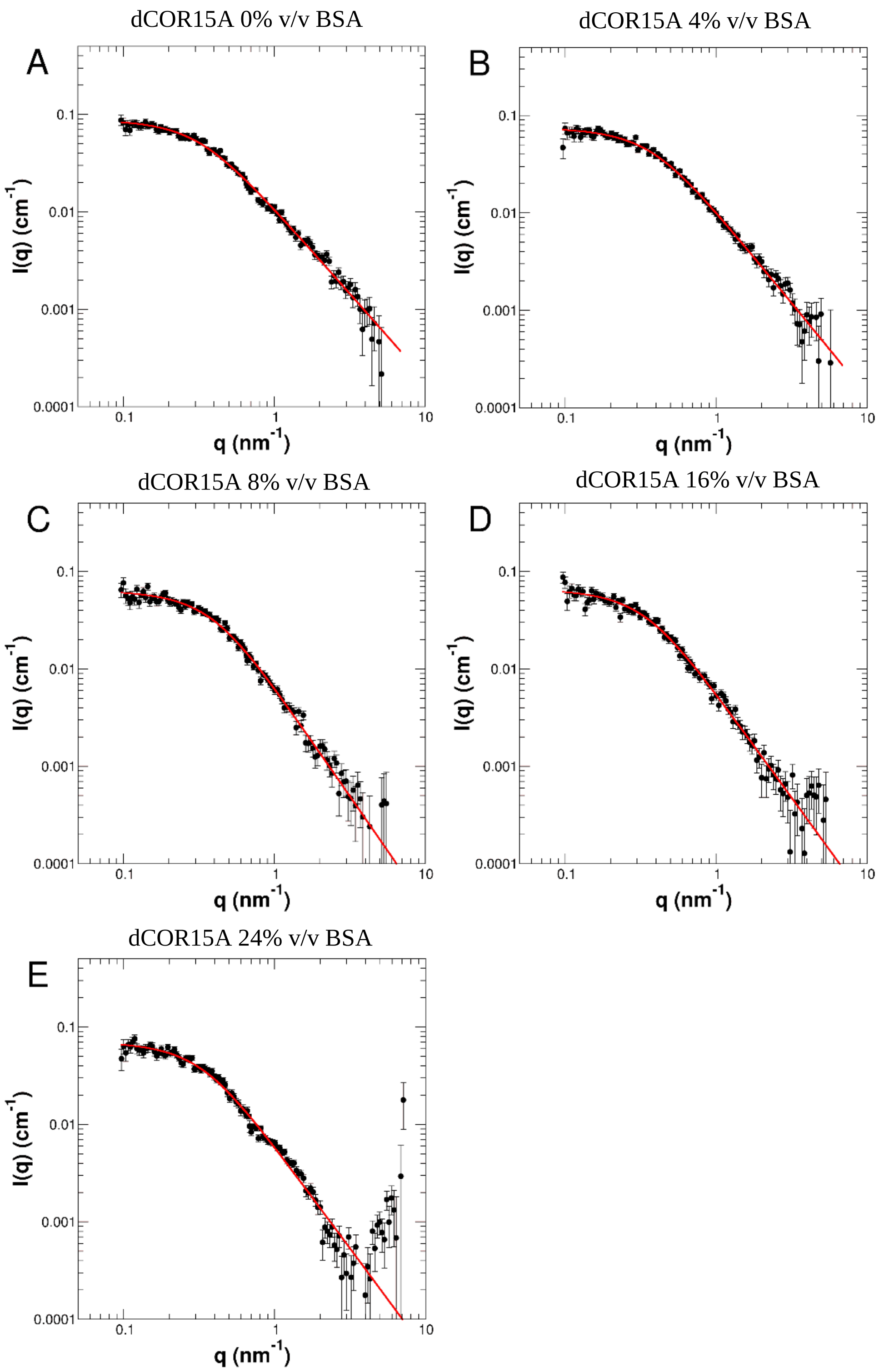

Fig. S1: Neutron scattering data of COR15A in increasing volume fractions of the proteinaceous crowder BSA. The symbols represent the experimental SANS data in log–log representation with solid lines depicting fits using generalized Gauss functions.

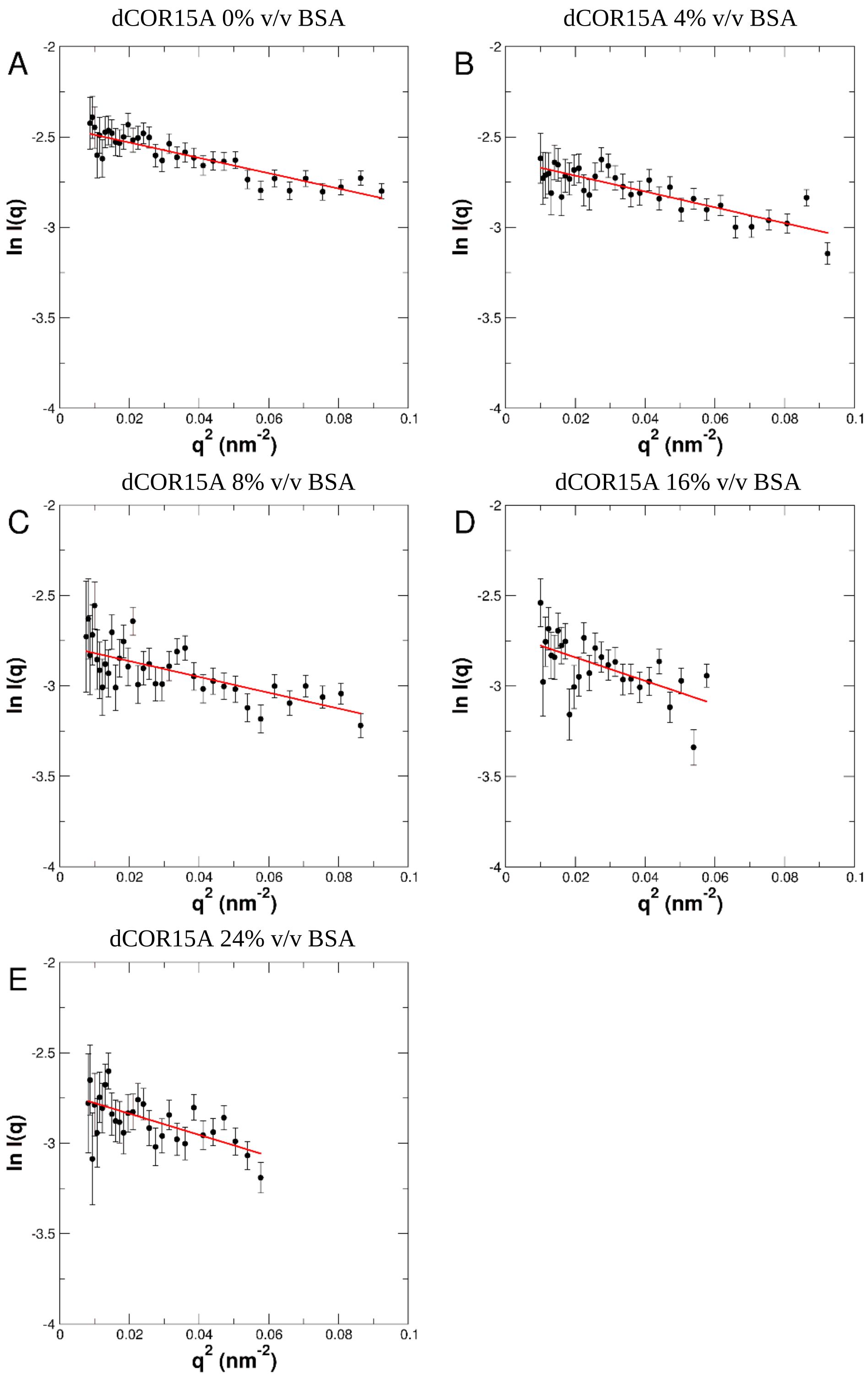

Fig. S2: Neutron scattering data of COR15A in increasing volume fractions of the proteinaceous crowder BSA. The symbols represent the SANS data at low q angles in the form of Guinier plots, with solid lines depicting fits, which were used for calculation of R_G.

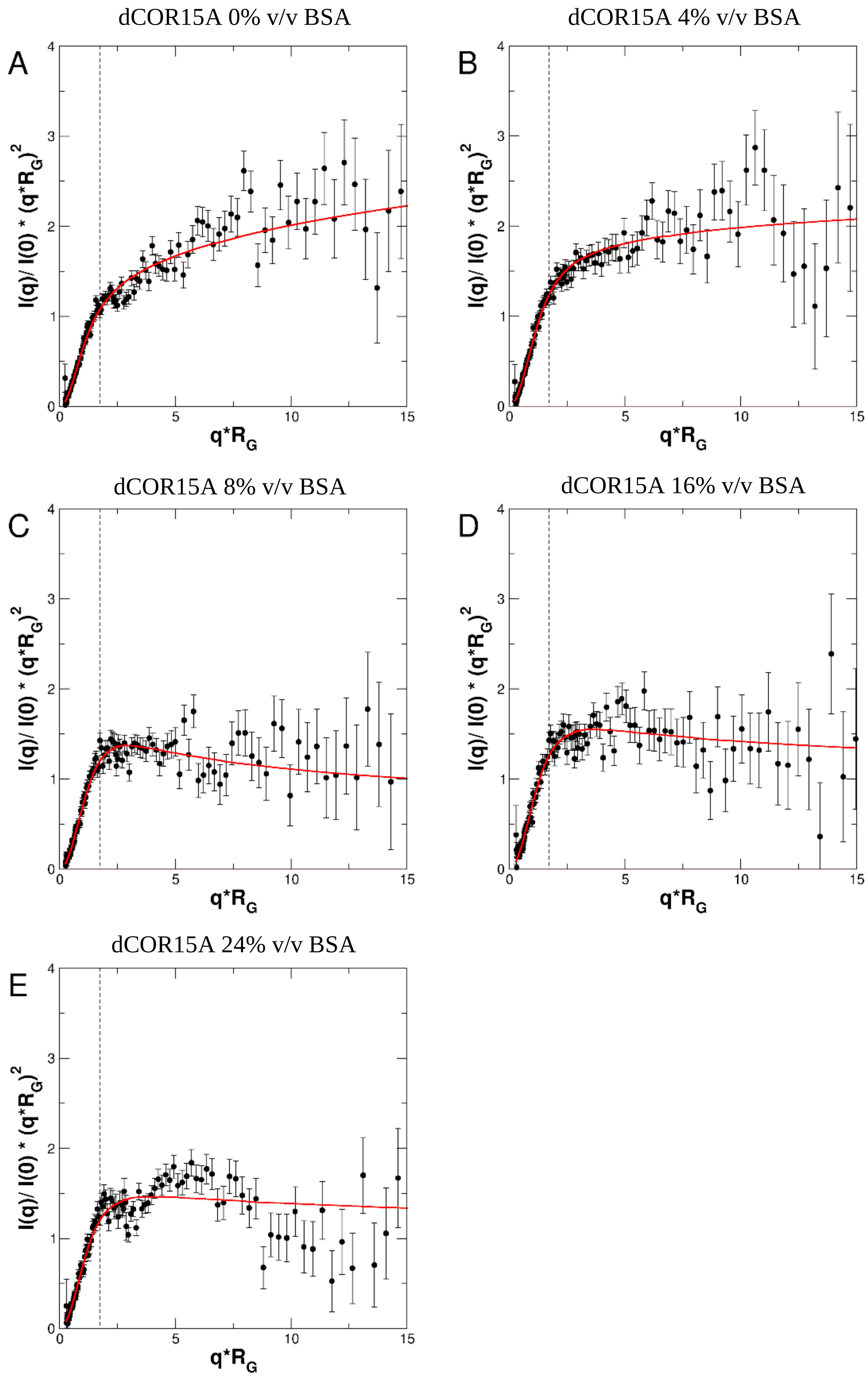

Fig. S3: Neutron scattering data of COR15A in increasing volume fractions of the proteinaceous crowder BSA expressed as normalized Kratky plots. The dashed lines indicate a peak position at $qR_G = 3^{0.5}$, which is expected for a folded globular protein.

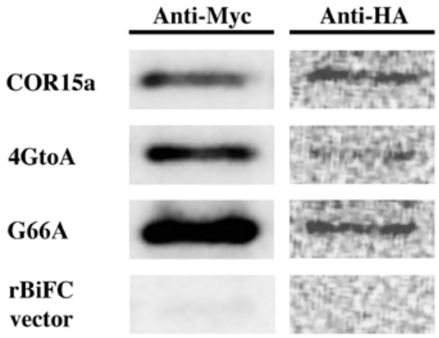

Fig. S4: Expression controls of plants carrying either the COR15A, 4GtoA or G66A sequence in expression sites 1 and 2 and the empty vector as a negative control, referring to BiFC data in Fig. 2. Both putative interaction partners in the raw protein lysates were immunodetected with anti-HA and anti-Myc antibody, respectively.

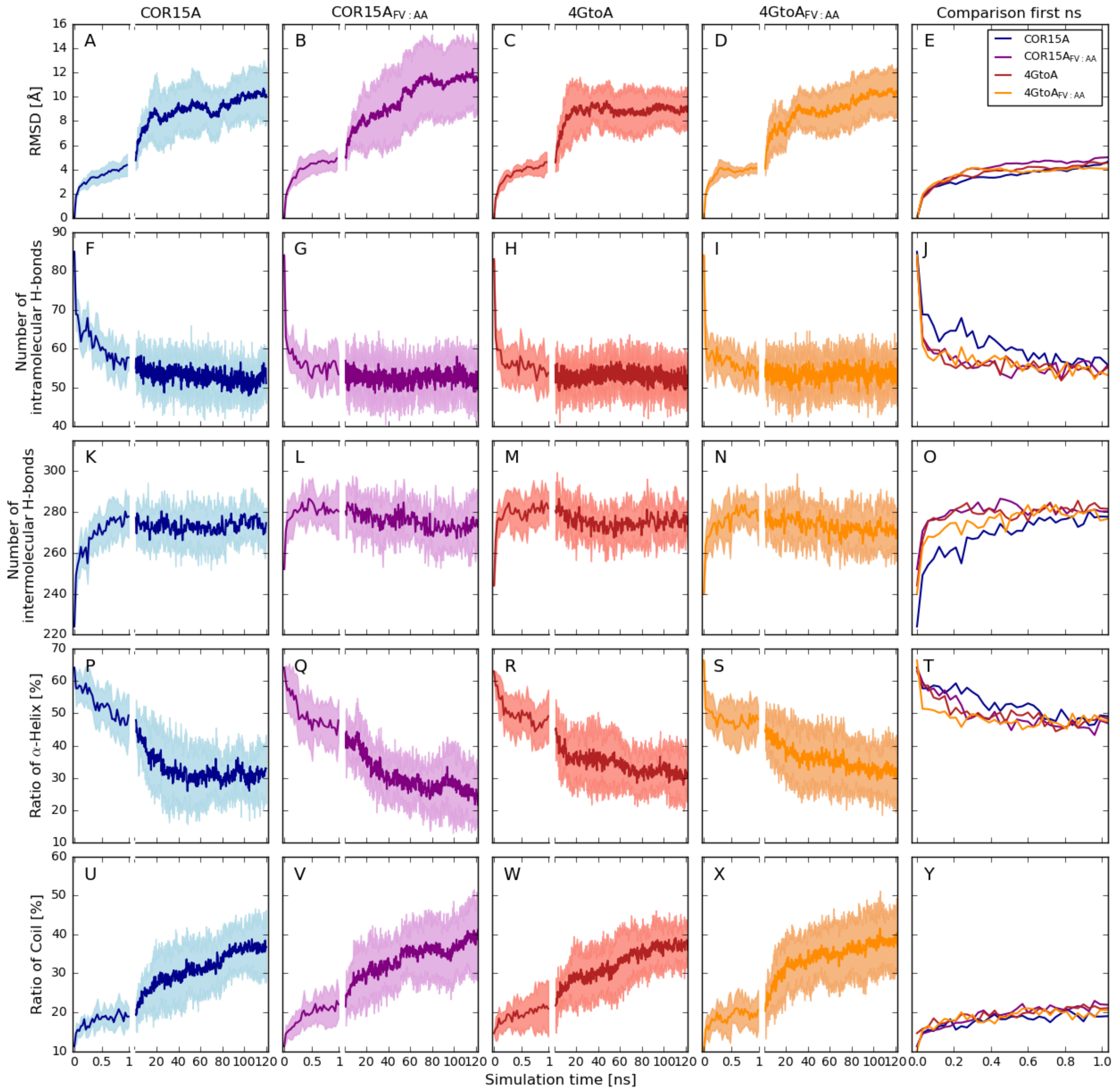

Fig. S5: RMSD (A-D), intra- (F-I) and intermolecular (K-N) H-bonds and secondary structure in terms of the ratio of random coil (P-S) and α-helix (U-X) of the monomeric COR15A variants during 120 ns MD simulations. Error bars depict the standard deviation of 10 simulation repeats. (E), (J), (O), (T) and (Y) show the average slopes of the respective parameter during the first ns of MD simulation for all COR15A variants in direct overlay.

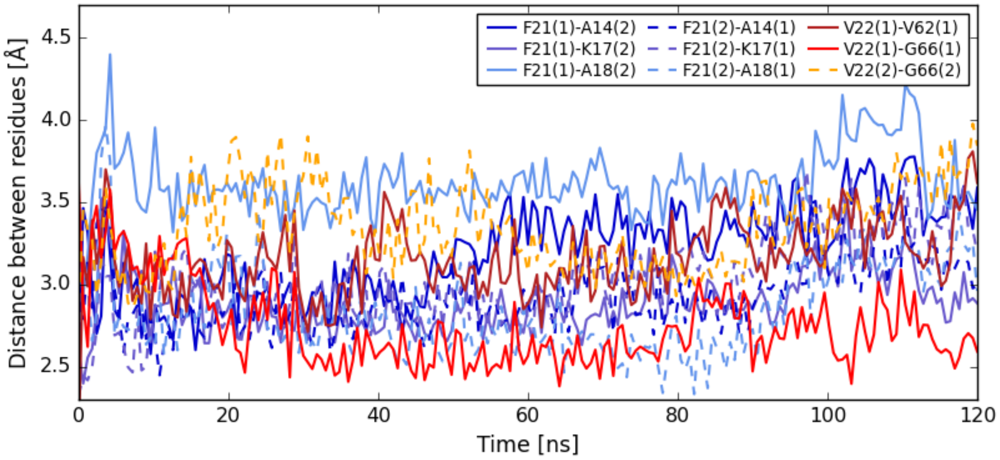

Fig. S6: Distance between the residues involved in most contacts as depicted in Fig. 3J is stable throughout simulation time

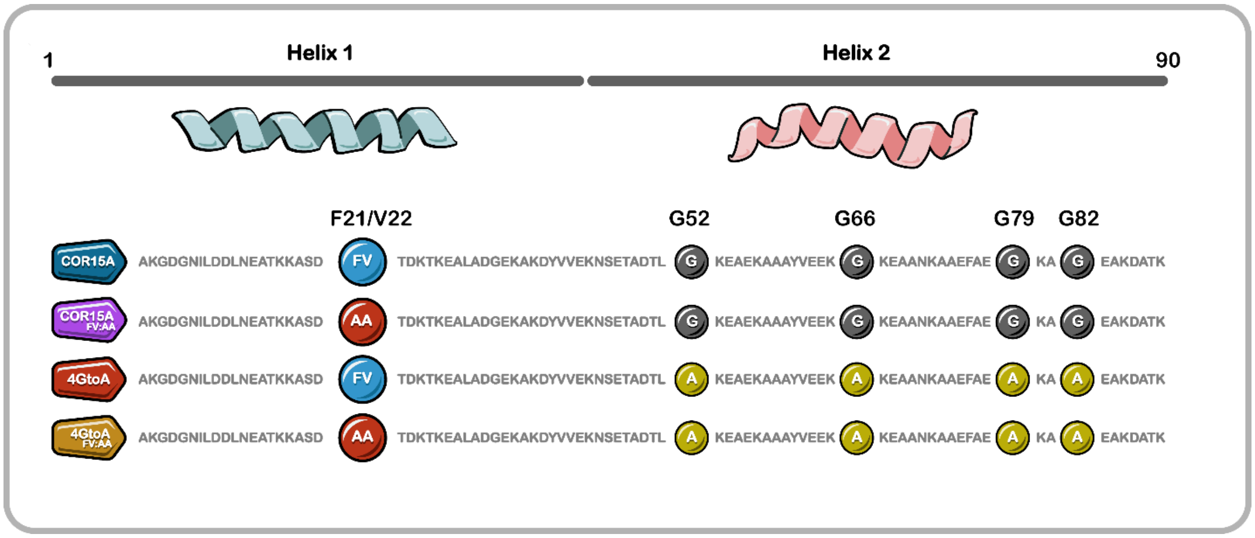

Fig. S7: Sequences of COR15A WT and mutants investigated in this study. The 4GtoA mutation is indicated in grey and yellow colors and the FVAA mutation in blue and red colors. The two helical domains as predicted in the docked COR15A dimer model are indicated and referred to as helix 1 (H1) and helix 2 (H2).

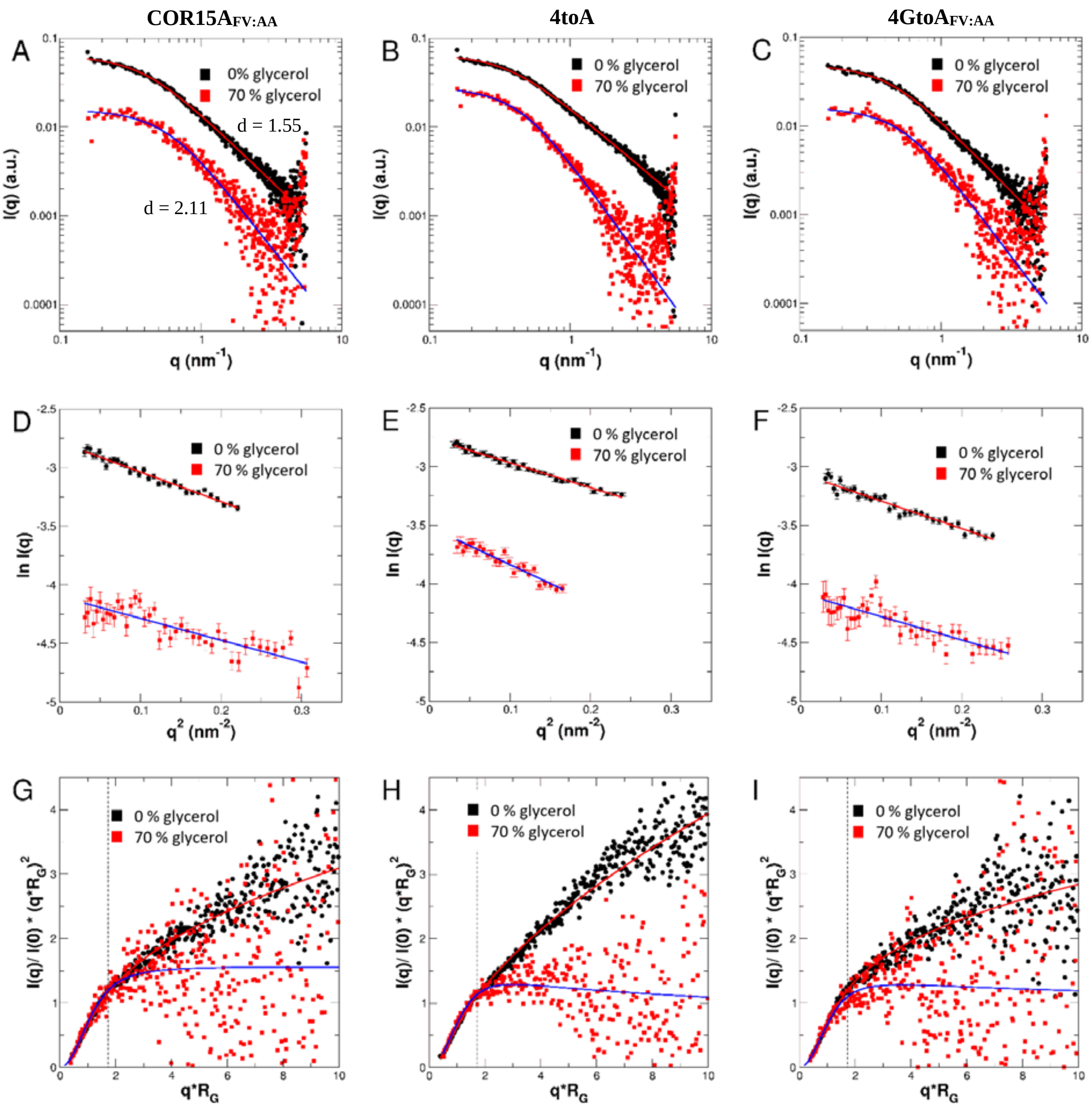

Fig. S8: X-ray scattering data of COR15A variants without glycerol (black symbols) and at 70 % glycerol (red symbols). The symbols represent the experimental data. The respective data for COR15A have been published previously (1). (A-C) show the SAXS data in log–log representation with solid lines depicting fits using generalized Gauss functions. (D-F) represent the SAXS data at low q angles in the form of Guinier plots, which were used for calculation of R_G. (G-I) express the SAXS data as normalized Kratky plots. The dashed lines indicate a peak position at $qR_G = 3^{0.5}$, which is expected for a folded globular protein.

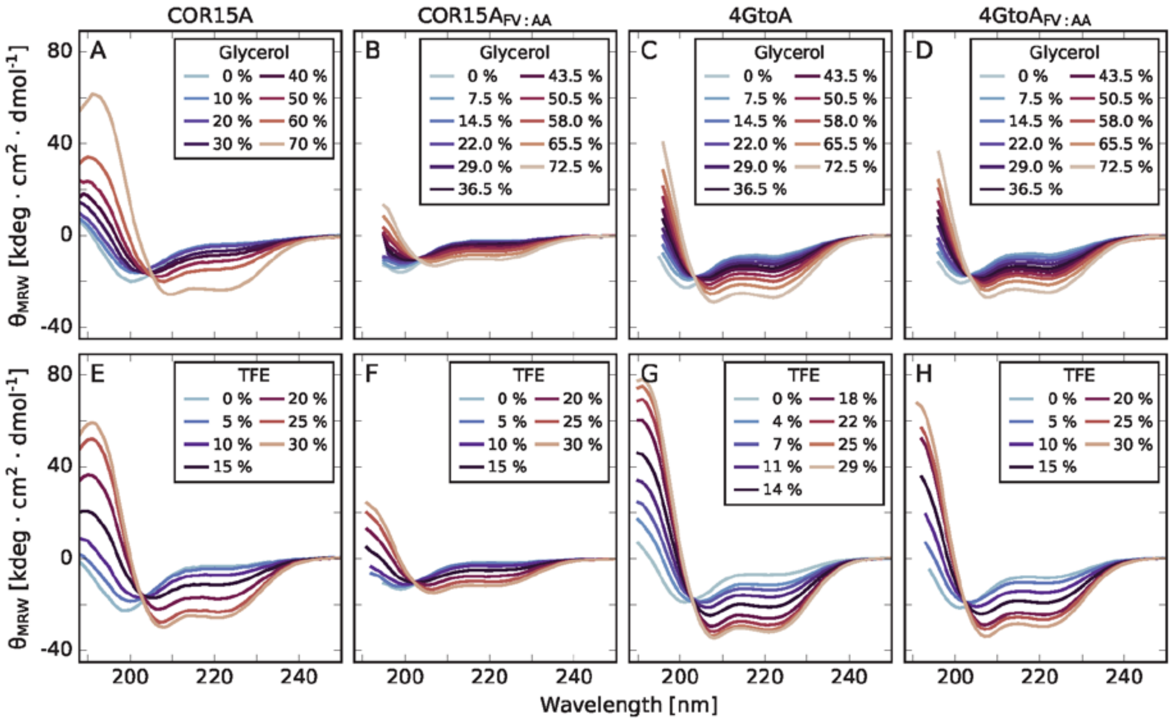

Fig. S9: Far-UV CD spectra of COR15A and its mutants in increasing concentrations of the cosolutes glycerol and TFE

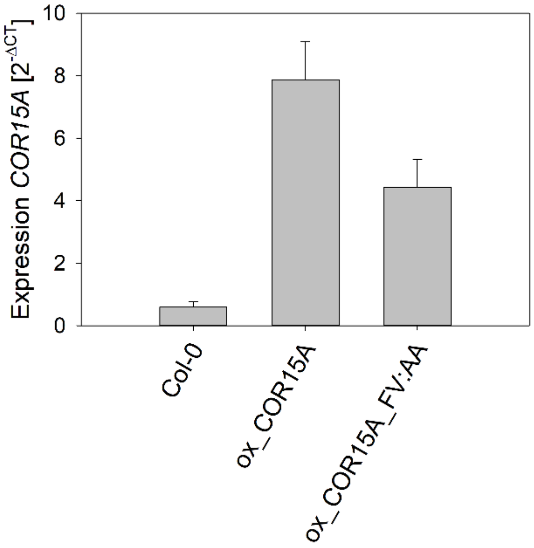

Fig. S10: Relative expression levels of the *COR15A* gene in plants used for electrolyte leakage experiments as measured by qPCR. Expression was normalized to the average of the two housekeeping genes actin and GAPDH ($2^{-\Delta CT}$).

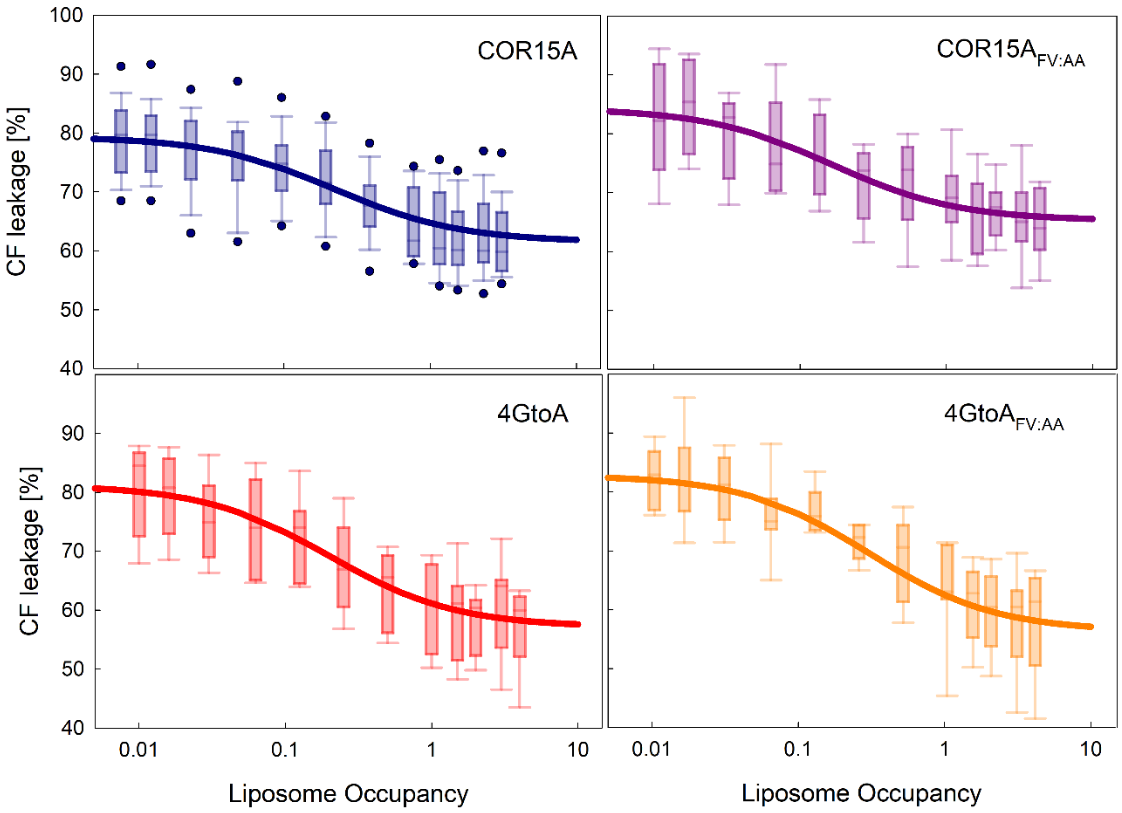

Fig. S11: CF leakage from ICMM liposomes after a freeze-thaw cycle to -20 °C. ICMMs were frozen in increasing concentrations of COR15A and its mutants at 12 different liposome surface occupancies. Boxes are plotted as a function of ICMM surface occupancy in order to account for size differences between the COR15A variants and depict median and quartiles. Whiskers represent 10 and 90% percentiles and the symbols 5 and 95% percentiles. Solid lines depict the regression curves from fitting the raw data to a dose response model as shown in Fig. 6B.

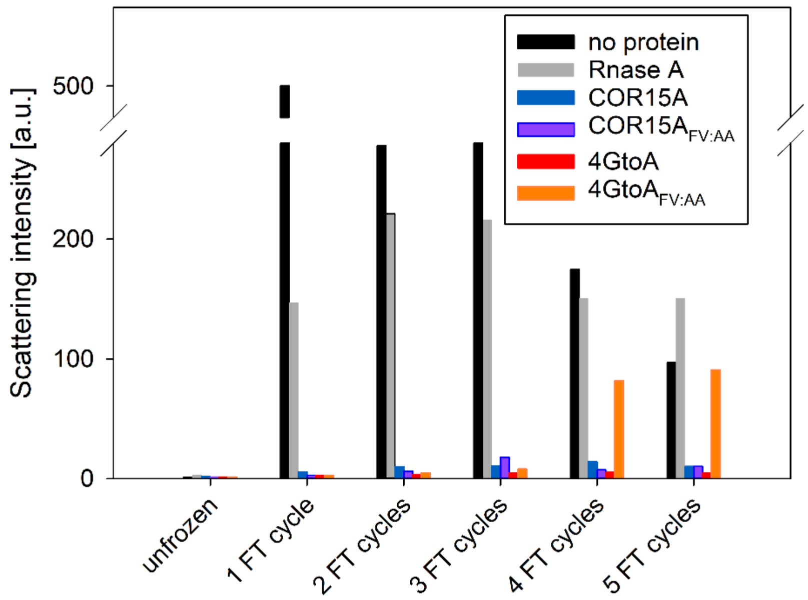

Fig. S12: Light scattering intensities of 0.15 g/L LDH in 30 mM sodium phosphate buffer, pH 7.4 alone or in the presence of the COR15A variants or the control protein RNase A in concentrations referring to LDH surface occupancies of 4-5 after zero to five freeze-thaw cycles in liquid nitrogen. Scattering intensities were measured in a custom built light scattering instrument and normalized to toluene as internal reference.

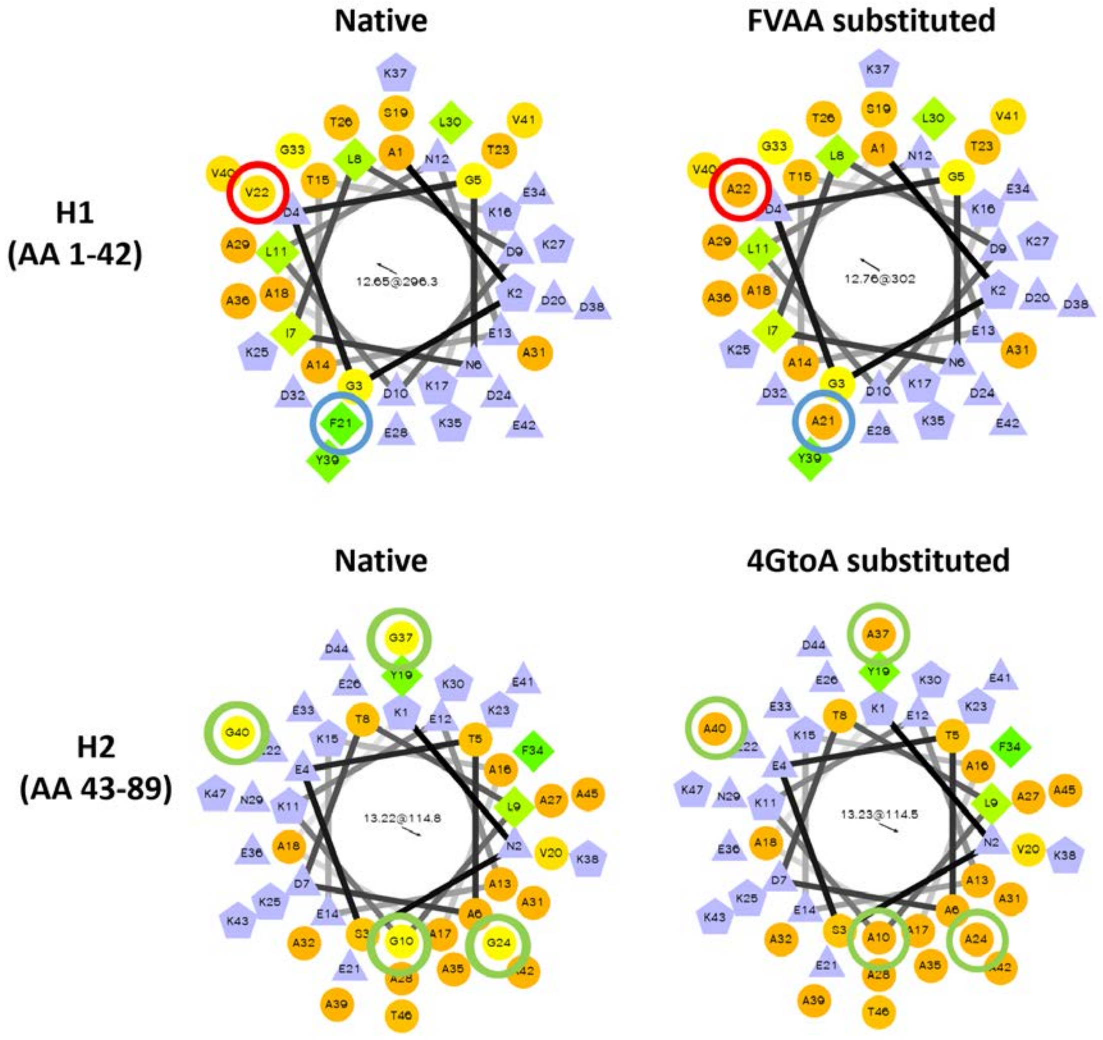

Fig. S13: Helical wheel projections of H1 (upper row) and H2 (lower row) from COR15A in the native forms (left) and after introduction of the respective mutations (right) (2). Helical wheels were drawn with https://pss.sjtu.edu.cn/cgi-bin/wheel.cgi using 3.6 residues per helix turn. Numbering of amino acids in H2 starts from 1 for technical reasons. Encircled amino acids represent sites of amino acid substitutions, with blue representing position 21, red position 22 (both on H1) and green positions 10, 24, 37 and 40 on H2, corresponding to 52, 66, 79 and 82 in the full length sequence. Arrows and numbers in the helix center represent magnitude and direction of the hydrophobic moment (µH).

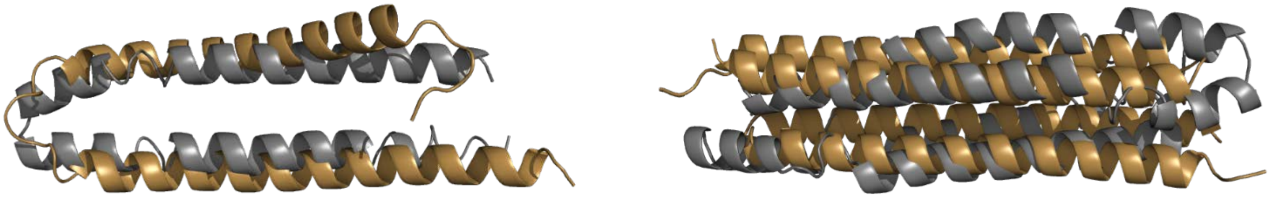

Fig. S14: Overlay of monomer (left) and dimer (right) structures of COR15A modelled and docked by I-TASSER and Haddock (silver) and AlphaFold (gold). The RMSD between monomers and dimers modelled by I-TASSER and AF was 5.3 A and 6.9 A, respectively.

Construct	Expression cassette	Purpose	Exemplary pictures				
			Brightfield	RFP	Chlorophyll	YFP	Overlay
pBiFCt-2in1-CC-COR15A/COR15A	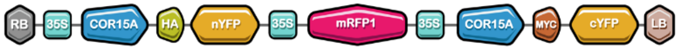	rBIFC/ Co-IP	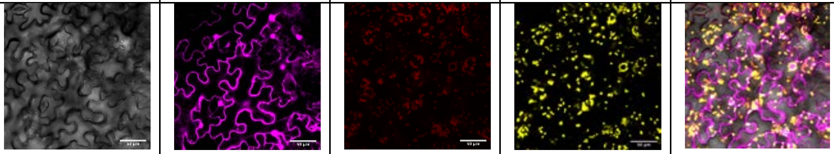				
pBiFCt-2in1-CC-G66A/G66A	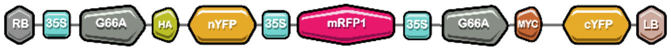	rBIFC	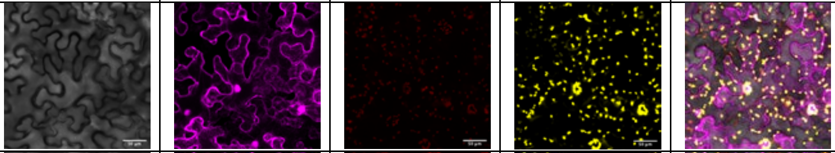				
pBiFCt-2in1-CC-4GtoA/4GtoA	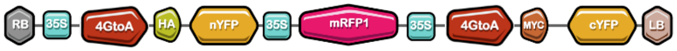	rBIFC	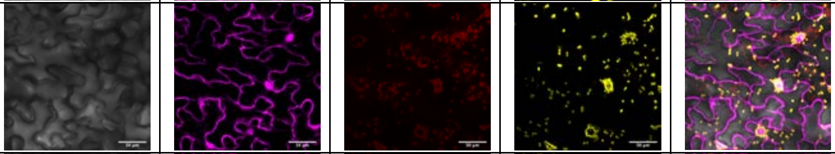				
pBiFCt-2in1-CC-COR15A$_{FV:AA}$/ COR15A$_{FV:AA}$	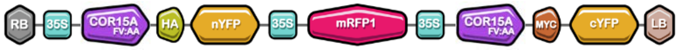	rBIFC/ Co-IP	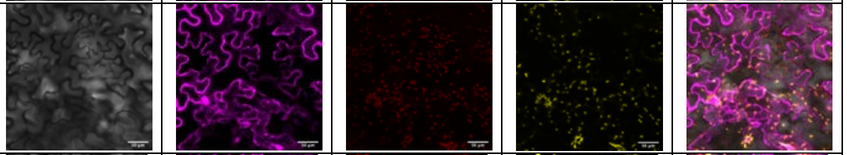				
pBiFCt-2in1-CC-4GtoA$_{FV:AA}$/ 4GtoA$_{FV:AA}$	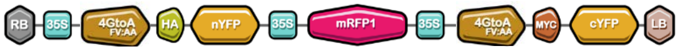	rBIFC/	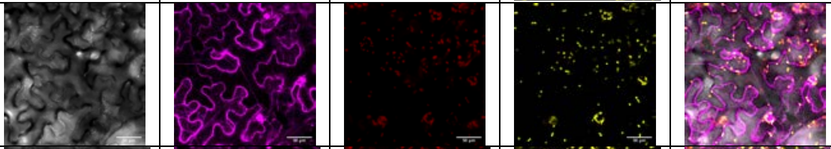				
pBiFCt-2in1-CC-COR15B/COR15A	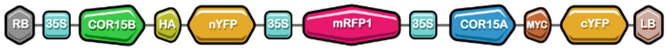	rBIFC/ Co-IP	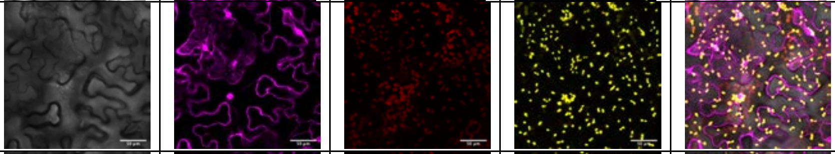				
pBiFCt-2in1-CC-COR15A/COR15B	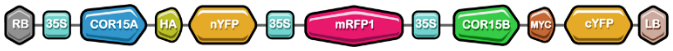	rBIFC/ Co-IP	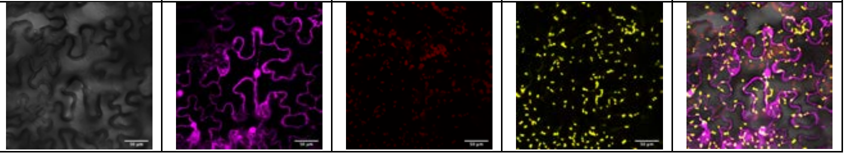				

pBiFCt-2in1-CC-COR15B/ COR15A$_{FV:AA}$	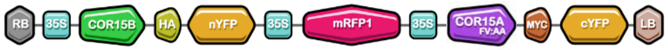	rBIFC/ Co-IP	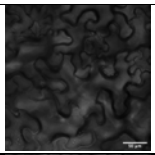	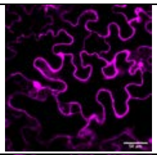	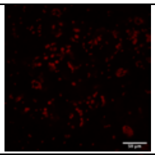	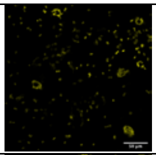	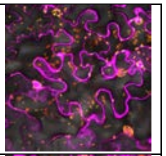
pBiFCt-2in1-CC-COR15A$_{FV:AA}$ /COR15B	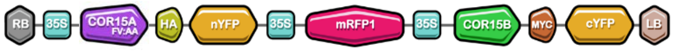	rBIFC/ Co-IP	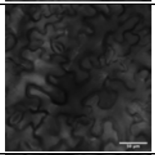	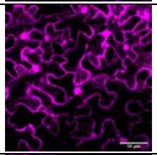	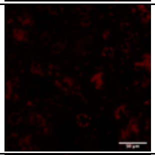	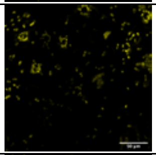	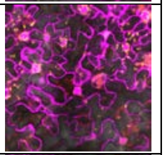
pBiFCt-2in1-CC-COR15A-SP/ COR15A-SP	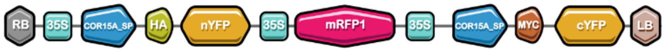	rBIFC/ Co-IP	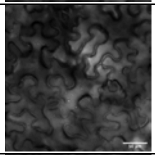	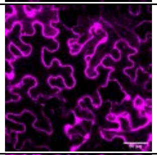	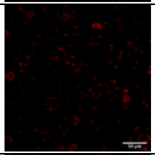	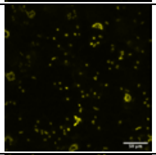	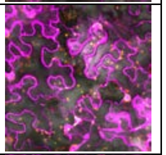
pBiFCt-2in1-CC-COR15B-SP/ COR15B-SP	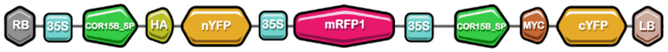	rBIFC/ Co-IP	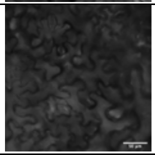	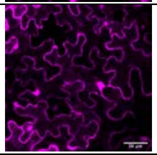	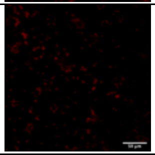	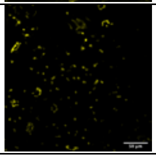	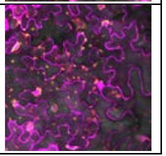

Fig. S15: Exemplary confocal pictures of Tobacco leaves transiently expressing plasmids containing the constructs used for rBiFC and Co-IP.

Table S1: List of cloning primers

Primer Name	5'-3' sequence	Application
COR15A ORF fw	ATGGCGATGTCTTTCTCAGGAG	sequencing
COR15A ORF Rv	CTACTTTGTGGCATCCTTAGCC	cloning (pMDC32)
COR15B ORF Fw	ATGGCGATGTCTTTATCAGGAG	sequencing
COR15B ORF Rv	GGGACTTTGTGGCATTCTTAGC	sequencing
pENTR-COR15A Fw	CATGGCGATGTCTTTCTCAGGAG	cloning (pMDC32)
COR15A Fw attB1	GGGGACAAGTTTGTACAAAAAAGCAGGCTTAATGGCGATGTCTTTCTCAGGA	cloning (rBiFC)
COR15A Rv attB4	GGGGACAACTTTGTATAGAAAAGTTGGGTGCTTTGTGGCATCCTTAGCCT	cloning (rBiFC)
COR15A Fw attB3	GGGGACAACTTTGTATAATAAAGTTGGAATGGCGATGTCTTTCTCAGGA	cloning (rBiFC)
COR15A Rv attB2	GGGGACCACTTTGTACAAGAAAGCTGGGTGCTTTGTGGCATCCTTAGCCT	cloning (rBiFC)
COR15B Fw attB1	GGGGACAAGTTTGTACAAAAAAGCAGGCTTAATGGCGATGTCTTTATCAGGAG	cloning (rBiFC)
COR15B Rv attB4	GGGGACAACTTTGTATAGAAAAGTTGGGTGGGACTTTGTGGCATTCTTAGC	cloning (rBiFC)
COR15B Fw attB3	GGGGACAACTTTGTATAATAAAGTTGGAATGGCGATGTCTTTATCAGGAG	cloning (rBiFC)
COR15B Rv attB2	GGGGACCACTTTGTACAAGAAAGCTGGGTGGGACTTTGTGGCATTCTTAGC	cloning (rBiFC)
qRT-COR15A Fw	TGTCAGAGTCGGCCAGAAAAC	qRT-PCR
qRT-COR15A Rv	CACCTTTAGCGGCGTAGATCA	qRT-PCR
HA-tag Rv	GCATAATCAGGAACATCATAAGG	sequencing rBiFC
Myc-tag Rv	CTGAGATAAGCTTCTGTTCCATT	sequencing rBiFC
COR15A-SP attb1	GGGGACAAGTTTGTACAAAAAAGCAGGCTTAATGGCGATGTCTTTCTCAGGAG	cloning (rBiFC)
COR15A-SP attb4	GGGGACAACTTTGTATAGAAAAGTTGGGTGGTAGATCAACGACTTCTTGCGTT	cloning (rBiFC)
quickchange FV:AA	TTTTATCCGTCGCCGCATCTGAA	Mutant generation
quickchange FV:AA	GCTTCAGATGCGGCGACGGATAA	Mutant generation
quickchange 4GtoA	CCAGCGATGCGGCGACCGATAAA	Mutant generation
quickchange 4GtoA	TTTTATCGGTCGCCGCATCGCTG	Mutant generation
NOS-T Rv	CTTTATTGCCAAATGTTTGAACG	sequencing (pMDC32)

Table S2: List of plasmid constructs

**Construct name**	**Application**
pENTRD::COR15Aa ORF	Subcloning
pENTRD::4GtoA	Subcloning
pENTRD::COR15A$_{FV:AA}$	Subcloning
pMDC32::COR15A	Arabidopsis transgenic lines/freezing experiments
pMDC32::4GtoA	Arabidopsis transgenic lines/freezing experiments
pMDC32::COR15A$_{FV:AA}$	Arabidopsis transgenic lines/freezing experiments
pProExhbt::FV:AA	protein expression/ *in vitro* characterization
pProExhbt::4GtoA$_{FV:AA}$	Protein expression/ *in vitro* characterization
pDONR-L3L2::COR15a-SP	Subcloning
pDONR-L3L2::4GtoA	Subcloning
pDONR-L3L2::G66A	Subcloning
pDONR-L3L2::COR15A$_{FV:AA}$	Subcloning
pDONR-L1L4::COR15A	Subcloning
pDONR-L1L4::COR15A$_{FV:AA}$	Subcloning
pDONR-L1L4::4GtoA	Subcloning
pDONR-L1L4::G66A	Subcloning
pDONR-L3L2::COR15A-SP	Subcloning
pDONR-L1L4::COR15A-SP	Subcloning
pDONR-L3L2::COR15B	Subcloning
pDONR-L1L4::COR15B	Subcloning
pDONR-L3L2::COR15B-SP	Subcloning
pDONR-L1L4::COR15B-SP	Subcloning
pDONR-L3L2::COR15B-SP	Subcloning
pDONR-L1L4::COR15B-SP	Subcloning
pBiFCt-2in1-CC-COR15A/COR15A	rBiFC/Co-IP (see Fig. S15)
pBiFCt-2in1-CC-G66A/G66A	rBiFC (see Fig. S15)
pBiFCt-2in1-CC-4GtoA/4GtoA	rBiFC (see Fig. S15)
pBiFCt-2in1-CC-COR15A$_{FV:AA}$/COR15A$_{FV:AA}$	rBiFC/Co-IP (see Fig. S15)
pBiFCt-2in1-CC-4GtoA$_{FV:AA}$/4GtoA$_{FV:AA}$	rBiFC (see Fig. S15)
pBiFCt-2in1-CC-COR15B/COR15A	rBiFC/Co-IP (see Fig. S15)
pBiFCt-2in1-CC-COR15A/COR15B	rBiFC/Co-IP (see Fig. S15)
pBiFCt-2in1-CC-COR15B/COR15A$_{FV:AA}$	rBiFC/Co-IP (see Fig. S15)
pBiFCt-2in1-CC-COR15A$_{FV:AA}$/COR15B	rBiFC/Co-IP see Fig. S15)
pBiFCt-2in1-CC-COR15A-SP/COR15A-SP	rBiFC/Co-IP (see Fig. S15)
pBiFCt-2in1-CC-COR15B-SP/COR15B-SP	rBiFC/Co-IP (see Fig. S15)

SI References

1. K. Shou *et al.*, Conformational selection of the intrinsically disordered plant stress protein COR15A in response to solution osmolarity – an X-ray and light scattering study. *Phys. Chem. Chem. Phys.* **21**, 18727-18740 (2019).
2. R. Zidovetzki, B. Rost, D. L. Armstrong, I. Pecht, Transmembrane domains in the functions of Fc receptors. *Biophys. Chem* **100**, 555-575 (2003).
